# Synthesis, Characterization, Thermal Analysis, DFT, and Cytotoxicity of Palladium Complexes with Nitrogen-Donor Ligands

**DOI:** 10.3390/molecules27030964

**Published:** 2022-01-31

**Authors:** Sattar R. Majeed, Mina A. Amin, Fawzy A. Attaby, Marta E. Alberto, Ahmed A. Soliman

**Affiliations:** 1 Department of Chemistry, Faculty of Science, Cairo University, Giza 12613, Egypt; sc.sat70tar@uoanbar.edu.iq (S.R.M.); mina@sci.cu.edu.eg (M.A.A.); fattaby@sci.cu.edu.eg (F.A.A.); 2 Department of Chemistry, College of Science, University of Anbar, Ramadi 30001, Iraq; 3 Department of Chemistry and Chemical Technologies, University of Calabria, 87036 Arcavacata di Rende, CS, Italy

**Keywords:** palladium complex, dinitrogen ligands, cytotoxicity, hydrolysis, DFT, TDDFT

## Abstract

Three new palladium complexes ([Pd(DABA)Cl_2_], [Pd(CPDA)Cl_2_], and [Pd(HZPY)Cl_2_]) bearing dinitrogen ligands (DABA: 3,4-diaminobenzoic acid; CPDA: 4-chloro–o-phenylenediamine; HZPY: 2-hydraziniopyridine) were synthesized, characterized, and tested against breast cancer (MCF-7), prostate carcinoma cell line (PC3) and liver carcinoma cell line (HEPG2). [Pd(DABA)Cl_2_] complex exhibited the highest inhibition percentage, lying between 68–71%. The hydrolysis mechanism of each palladium complex, the key step preceding the binding to the biological target, as well as their photophysical properties were explored by means of DFT and TDDFT computations. Results indicate a faster hydrolysis process for the Pd(DABA)Cl_2_ complex. The computed activation energies for the first and second hydrolysis processes suggest that all the compounds could reach DNA in their monohydrated form.

## 1. Introduction

**Figure 1:**
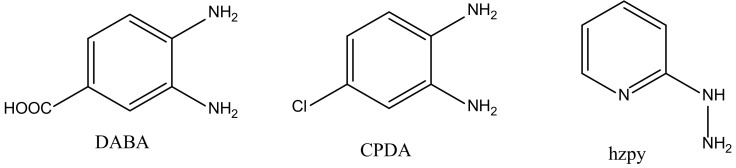
Dinitrogen ligands DABA, CPDA, and hzpy used in the preparation of palladium complexes.

Coordination chemistry provides a massive number of metal complexes that can be used in different biological applications, including therapeutic and diagnostic medicine. [1,2,3,4,5,6,7,8]. Cisplatin (*cis*-dichlorodiammineplatinum(II), *cis*-Pt(Cl_2_(NH_3_)_2_) represents one of the great success stories in the field of cancer chemotherapy for the treatment of testicular tumors whose biological activity arises from its ability to covalently bind DNA, inhibit transcription and replication, and ultimately cause cell death or apoptosis [9,10]. For over three decades, continuous efforts have been made in order to overcome the drawbacks of cisplatin, which include nephrotoxicity, neurotoxicity, ototoxicity, and myelosuppression, with a primary focus on the development of new derivatives with improved pharmacological properties and fewer side effects [11,12]. Palladium was chosen as an alternative for platinum since they share similar chemical behaviors and the possibility to form square planar complexes. Pd(II) complexes are kinetically and thermodynamically stable and are 10^5^ more reactive than analogues Pt(II) complexes. Nevertheless they display lower antitumor activities, probably due to their high reactivity that does not preserve the complex structure until it reach the DNA targets [13,14,15,16,17]. Pd(II) complexes based on nitrogen ligands attracted increasing attention due to their beneficial pharmacological properties [18]. Changing, modifying, and synthesizing novel/existing ligands can be potential ways of improving the biological activities and reducing drug resistance [18]. Tuning and improving the ligands’ design could also enhance the stability of the Pd(II) complexes through the use of chelating agents such as bidentate ligands [18,19,20,21]. Pd(II) complexes based on nitrogen-based ligands such as pyridine, quinolines, pyrazoles, and 1,10-phenanthroline have shown potential antitumor activities. These ligands stabilize and control the palladium-based species in the biological systems rather than reducing the cis-trans isomerism [22]. In the present article, we present a new series of Pd(II) complexes with potential cytotoxic activities. The palladium complexes were prepared using three bidentate ligands, namely, 3,4-diaminobenzoic acid (DABA), 4-chloro–o-phenylenediamine (CPDA), and 2-hydraziniopyridine hydrochloride (hzpy) and were spectroscopically and thermally characterized (Figure 1). The cytotoxic activities of the palladium complexes were studied against cancer cell lines. Moreover, to give insights on the hydrolysis reaction mechanism and kinetics, DFT studies are also herein presented together with TDDFT exploration of the UV–Vis spectra of each compound, providing characterizations of each transition band.

## 2. Results

### 2.1. Characterization of Palladium Complexes

#### 2.1.1. Spectral Data

##### [Pd(DABA)Cl_2_] Complex

**Figure 2:**
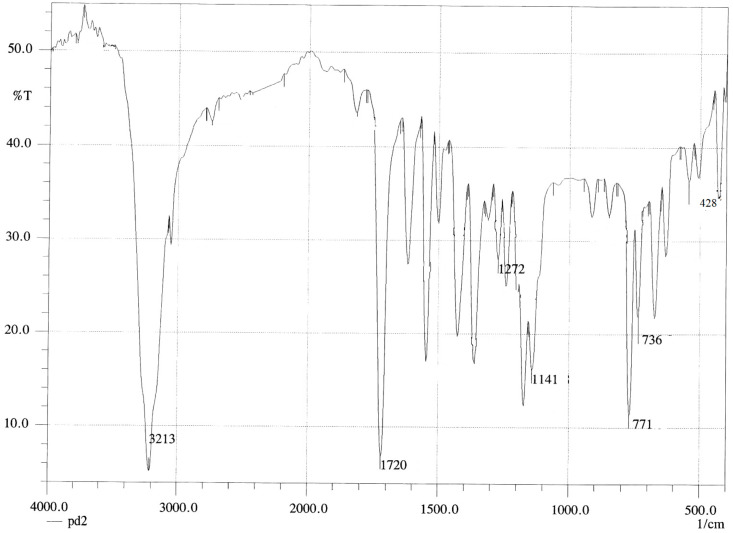
Experimental IR spectrum of the [Pd(DABA)Cl_2_] complex.

The IR spectrum of [Pd(DABA)Cl_2_] is shown in Figure 2.

The stretching vibrations of the amino groups of DABA are found at 3328 and 3209 cm^−1^ in the spectrum of the ligand (Figure S2a). These bands converge in one broad band shifted to a lower frequency (3213 cm^−1^) in the spectrum of the complex, suggesting the participation of the NH_2_ group in coordination. The involvement of the amino groups of the DABA ligand is also supported by the observation of the deformation vibrations of NH_2_, which are found at 1242 cm^−1^ (ρt NH_2_), 1172 cm^−1^ (ρwNH_2_), and 771 cm^−1^ (ρrNH_2_) (Table 1).

The band at 1624 cm^−1^ due to υC=O in the spectrum of the ligand (Figure S2a), appears shifted to higher frequencies in the complex spectrum (1720 cm^−1^). The shift of the υC=O of the carboxylic acid group to a higher wave number can be attributed to the liberation of the C=O from the intermolecular hydrogen bonding. The spectrum of the complex also shows an additional band at 428 cm^−1^ assigned to the M–N bond [23,24]. The mass spectral data is summarized in Table 2.

The mass spectrum of the [Pd(DABA)Cl_2_] complex (M. wt. = 329.48) gives the parent peak at *m*/*z* = 328 (M^+^-H), a peak at *m*/*z* = 193 (DABA-Cl-4H^+^), in addition to a peak at 141 assigned for (PdCl). The palladium isotopes also appeared at 110, 109, 108, 107, and 106. The magnetic susceptibility measurement of [Pd(DABA)Cl_2_] complex confirmed the diamagnetic nature of the complex, suggesting that the complex adopts a square planar structure and the Pd(II) (d^8^) center has the configuration of e_g_^4^ a_1g_^2^ b_2g_^2^ also confirmed by spectral and magnetic data, which prove that the DABA ligand acts as a bidentate ligand via the two amino groups.

##### [Pd(CPDA)Cl_2_] Complex

**Figure 3:**
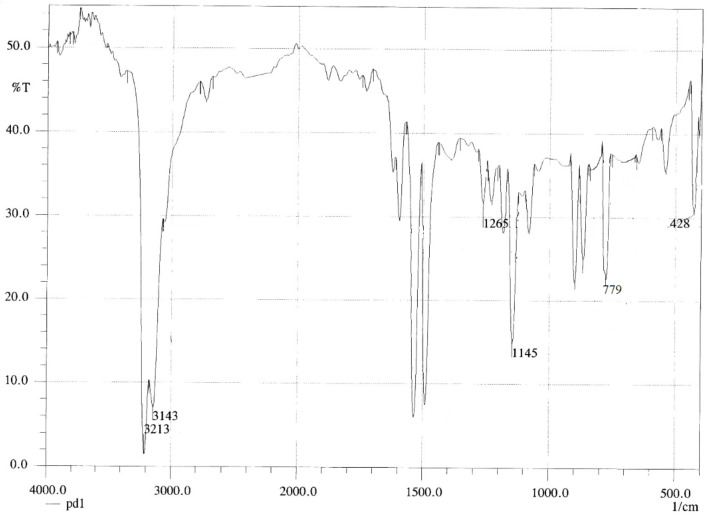
Experimental IR spectrum of the [Pd(CPDA)Cl_2_] complex.

The IR spectrum of [Pd(CPDA)Cl_2_] is shown in Figure 3.

The stretching vibrations of the amino groups of CPDA are found at 3402 and 3313 cm^−1^ (Figure S2b), while they appear as a strong peak shifted to lower frequencies (3213 and 3143 cm^−1^) in the spectrum of the complex, indicating chelation through the NH_2_ group. The deformation vibrations were found at higher frequencies, specifically 1265 cm^−1^ (ρt NH_2_), 1145 cm^−1^ (ρwNH_2_), and 779 cm^−1^ (ρrNH_2_), confirming the involvement of the NH_2_ in the coordination to the Pd(II) center (Table 1). The spectrum of the complex shows an additional band at 428 cm^−1^, assigned to the M–N bond [23,24]. The mass spectral data is summarized in Table 2. The mass spectrum of [Pd(CPDA)Cl_2_] complex (M. wt. = 319.91) gives a parent peak (M^+^) at *m*/*z* = 320 and two main peaks at *m*/*z* = 180, assigned for (CPDA-Cl_2_-4H^+^), and at 142, assigned for PdCl. The palladium isotopes appeared at 106, 107, 108, 109, and 110. The magnetic susceptibility measurement of the [Pd(CPDA)Cl_2_] complex confirms the diamagnetic nature of the complex and corroborates, together with the spectral and magnetic data, the square planar structure for [Pd(CPDA)Cl_2_], with a Pd(II) having a *d^8^* configuration (e_g_^4^ a_1g_^2^ b_2g_^2^) and with CPDA acting as a bidentate ligand via the two amino groups.

##### [Pd(hzpy)Cl_2_] Complex

**Figure 4:**
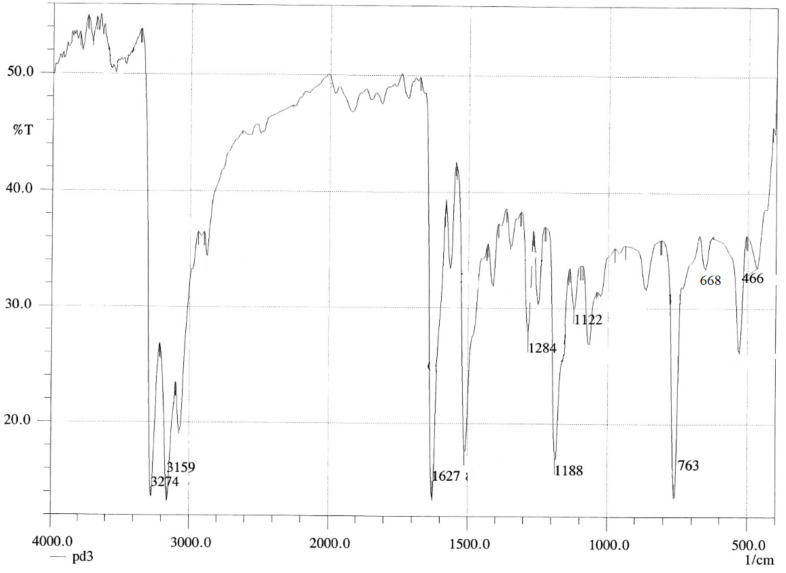
Experimental IR spectrum of the [Pd(hzpy)Cl_2_] complex.

The IR spectrum of [Pd(hzpy)Cl_2_] is shown in Figure 4. The stretching of the amino groups of hzpy found at 3305 and 3259 cm^−1^ in the spectrum of hzpy (Figure S2c) result in shifts to lower frequencies (3274 and 3159 cm^−1^) in the spectrum of the complex, confirming the complex formation upon the coordination of the amino group and pyridine nitrogen to the Pd ion. Another indicator of the coordination of the two amino groups to the Pd(II) ion is the shift of the deformation vibrations to higher frequencies at 1284 cm^−1^ (ρt NH_2_), 1188 cm^−1^ (ρwNH_2_), and 763 cm^−1^ (ρrNH_2_) (Table 1). The spectrum of the complex also shows an additional band at 466 cm^−1^ assigned to the M–N bond [23,24]. The mass spectral data is summarized in Table 2. The mass spectrum of [Pd(CPDA)Cl_2_] complex (M. wt. = 286.46) shows the parent peak (M^+^) at *m*/*z* = 286, in addition to other major peaks at *m*/*z* = 109 assigned to the hzpy ligand. An additional peak, belonging to (Cl_2_), appears at *m*/*z* = 71. The palladium isotopes appear at *m*/*z* = 109 and 110. The magnetic susceptibility measurement of the [Pd(hzpy)Cl_2_] complex confirm the diamagnetic nature of the complex, suggesting a square planar geometry with the *d^8^* configuration e_g_^4^ a_1g_^2^ b_2g_^2^ for the Pd(II) center. Spectral and magnetic data support such geometry for the [Pd(hzpy)Cl_2_] complex, with the hzpy ligand acting as a bidentate ligand via the NH_2_ group and nitrogen of the pyridine ring.

#### 2.1.2. ^1^HNMR Spectra

The ^1^HNMR spectra prove that the three palladium complexes are diamagnetic. For the [Pd(DABA)Cl_2_] complex, the aromatic protons of the DABA ligand are found at 7.69–8.33 ppm as multiplet, while the NH_2_ groups appear at 3.49 ppm. The aromatic protons, in the case of the [Pd(CPDA)Cl_2_] complex, appear at (7.69–8.33, m, 3H), and the NH_2_ groups at 4.79 ppm as a singlet peak. For the [Pd(hzpy)Cl_2_] complex, the aromatic protons of the hzpy ligand appear as multiplet (7.69–8.33, 3H), and the NH and NH_2_ group at 6.69 also appear as multiplet. There was a downfield shift in the peaks’ positions upon the formation of the three palladium complexes, confirming that the chelation process occurs via the NH_2_ groups [25].

### 2.2. Thermal Studies

The thermal stabilities of the palladium complexes were investigated through TGA and DTA graphs. The temperature ranges at which thermal decomposition was observed, along with the corresponding mass losses, are given in Table 3. Table 4 gives the decomposition temperature ranges, the DTG peak temperature, the correlation coefficients of the Arrhenius plots, as well as the thermodynamic parameters.

#### 2.2.1. Thermal Analysis of the [Pd(DABA)Cl_2_] Complex

**Figure 5:**
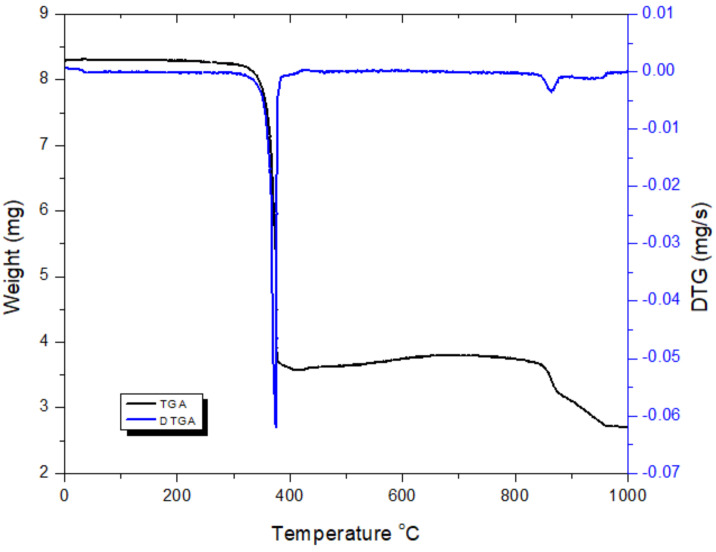
Thermogram of the [Pd(DABA)Cl_2_] complex (TG: black; DTG: blue).

**Scheme 1:**
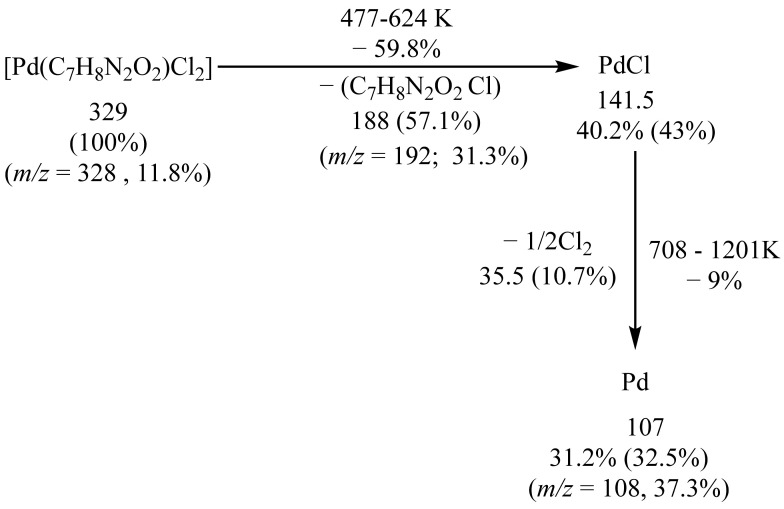
Thermal decomposition of the [Pd(DABA)Cl_2_] complex.

The thermogravimetric plot (Figure 5) shows that [Pd(DABA)Cl_2_] decomposes in two steps, as reported in Scheme 1. The weight losses were observed in the range 477–1201 K. The first decomposition peak (477–624 K) associated with the loss of (59.8%, calc. 57.1) is consistent with the elimination of DABA and 1/2 Cl_2_ species (*m*/*z* = 192). The second decomposition peak (708–1201 K), with a mass loss of (9.0%, calc. 10.7), is due to the loss of 1/2 Cl_2_ species, leaving Pd (31.2%, calc. 32.5%) as the metallic residue (*m*/*z* = 107.8).

#### 2.2.2. Thermal analysis of the [Pd(CPDA)Cl_2_] Complex

**Figure 6:**
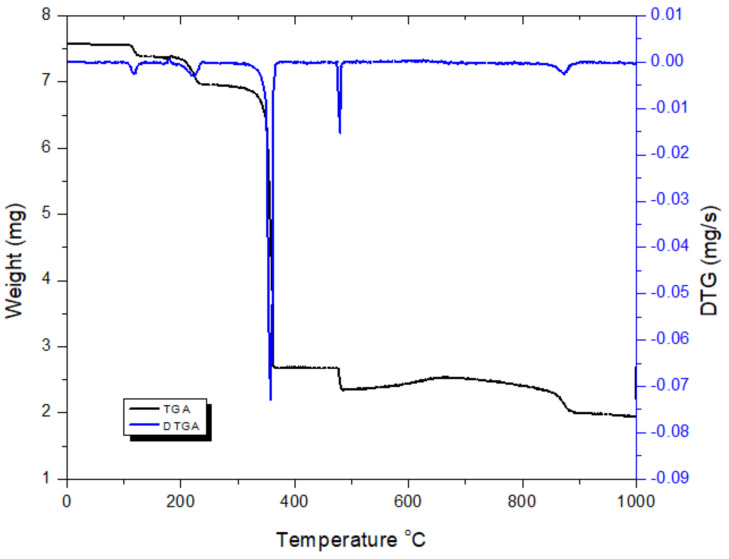
Thermogram of the [Pd(CPDA)Cl_2_] complex (TG: black; DTG: blue).

**Scheme 2:**
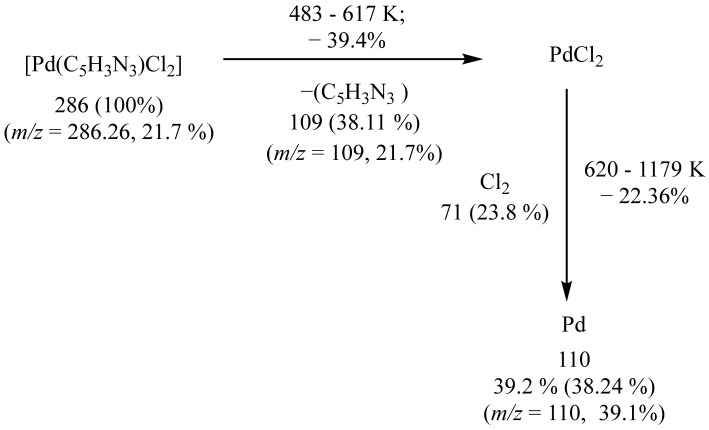
Thermal decomposition of the [Pd(CPDA)Cl_2_] complex.

The thermogravimetric plot of [Pd(CPDA)Cl_2_] (Figure 6) shows two decomposition steps, as reported in Scheme 2. The weight losses were observed in the temperature range 550–682 K. The first decomposition peak (550–682 K), with a mass loss of (56.4%, calc. 57.6%) is due to the loss of CPDA and 1/2 Cl_2_ species (*m*/*z* = 180). The second decomposition step (1057–1224 K), accompanied by the loss of (11.9%, calc. 11.09), could be attributed to the loss of 1/2 Cl_2_ species, leaving Pd metal (31.7%, calc. 33.06% as the metallic residue (*m*/*z* = 106.5).

#### 2.2.3. Thermal analysis of the [Pd(hzpy)Cl_2_] Complex

**Figure 7:**
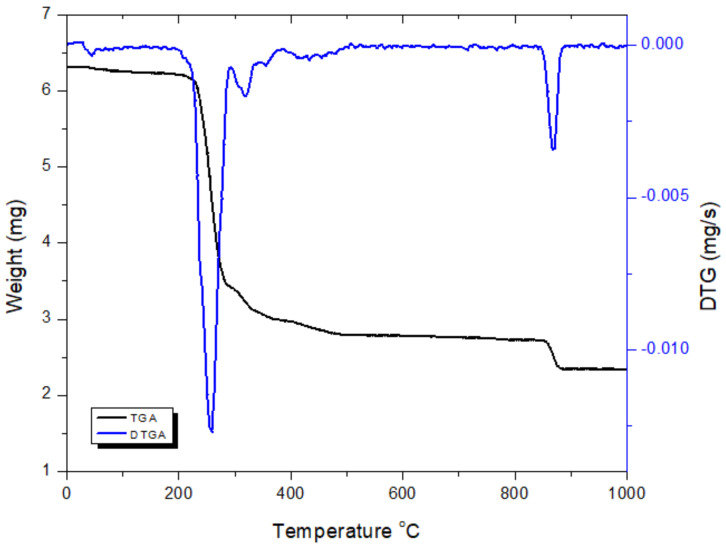
Thermogram of the [Pd(hzpy)Cl_2_] complex (TG: black; DTG: blue).

**Scheme 3:**
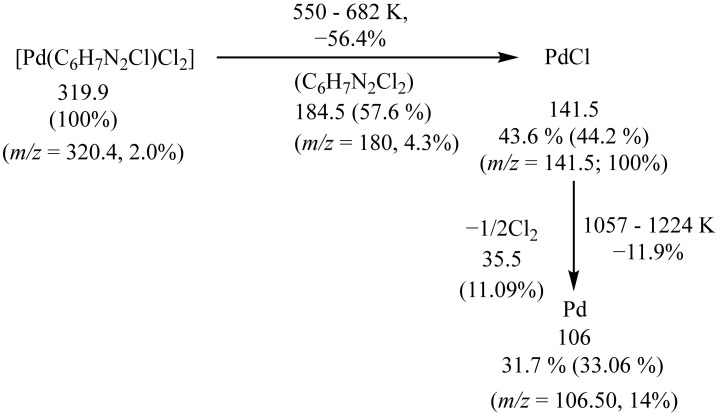
Thermal decomposition of the [Pd(hzpy)Cl_2_] complex.

Even the thermogravimetric plot of [Pd(hzpy)Cl_2_] (Figure 7) shows two decomposition steps (483–617 K), as reported in Scheme 3. The first decomposition peak (483–617 K) is linked to the loss of (49.4%, calc. 50.5%) and is consistent with the elimination of hzpy (*m*/*z* = 109). The second decomposition peak (620–1179 K) is connected to the loss of (22.36%, calc. 23.8%), and could be attributed to the loss of Cl_2_, leaving Pd metal (39.2%, calc. 38.24%) as the metallic residue (*m*/*z* = 110).

The thermodynamic parameters of the decomposition were calculated using Horowitz–Metzger and Coats–Redfern equations [26,27,28,29,30]. The correlation coefficients of the Arrhenius plots were found in the range from 0.62–0.98, indicating good fitness of the linear function. The three complexes are thermally stable with overall activation energy of 107, 573, and 580 kJ mol^–1^ for [Pd(DABA)Cl_2_], [Pd(CPDA)Cl_2_], and [Pd(hzpy)Cl_2_], respectively.

### 2.3. Theoretical Studies

**Figure 8:**
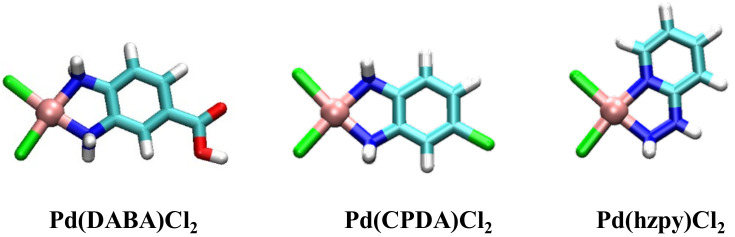
Optimized structures of the investigated complexes at the B3LYP/6-31+G(d,p)/SDD-level of theory, in water environment.

The optimized structures of the investigated compounds are displayed in Figure 8. The structural parameters of main interest, such as bond lengths and angles surrounding the metal center, are listed in Table S1, following the atom numbering defined therein.

An inspection of the computed parameters reveals that the Pd–N and Pd–Cl bond lengths are within the regular range reported in the literature [31], and the Pd metal center adopts a quite regular square planar conformation, with angles ranging between 90° and 95° between Pd and the chloride ions, and slightly smaller between Pd and the nitrogens of the bidentate ligands (81°–83°) (See Table S1).

**Figure 9:**
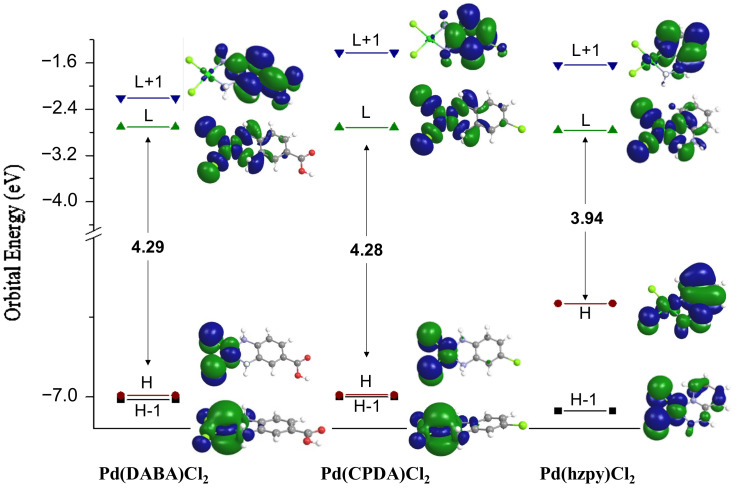
Energy diagram (eV) of the HOMO-1 (H-1), HOMO (H), LUMO (L), and LUMO+1 (L+1) orbitals for all the investigated compounds, also reporting the H–L energy gap (eV) and graphical molecular orbital plots.

Frontier molecular orbitals explorations (HOMO−1, HOMO, LUMO, LUMO+1) allow a better characterization of the ground-state electronic structures of these complexes. The energy diagram of the frontier MOs, with the indication of the HOMO–LUMO energy gaps and molecular orbitals plots, are reported in Figure 9. The main orbital composition (%) for each frontier orbital is reported in Figure S3.

A significant contribution of the metal characterizes the HOMO-1 orbitals of all the investigated compounds, being the Pd–d contribution comprised by 41 and 48%. The involvement of chloride ligands is also recognizable from the plots. The HOMO orbital is clearly metal-based for Pd(DABA)Cl_2_ and Pd(CPDA)Cl_2_ complexes, with an average Pd–d contribution of 46%. Interestingly, in the case of bidentate hzpy ligand, HOMO is clearly N–N ligand-based, with a consequent increase in energy of the orbital, and a consequent decrease of the HOMO–LUMO gap, as can be observed in Figure 8. Indeed, the LUMO orbital is predominantly based on the N–N ligands, with a degree of Pd–d mixing character similar for all the investigated compounds, and no changes in energy emerge with the choosing of the bidentate ligand, remaining quite constant for all the Pd compounds considered. Concerning the LUMO+1, an analysis of the orbital composition reveals that it is almost exclusively based on the bidentate ligand, and the metal contribution has almost vanished.

The inspection of the computed UV–Vis spectra of the investigated compounds (Figure S4) reveals the most intense absorption bands below 300 nm, which can be characterized as N–N ligand-centered for Pd complexes with DABA and CPDA, and assigned to a combination of mixed LC and LMCT characters for the Pd(hzpy)Cl_2_ complex, as shown by NTOs reported in Figure S5.

**Figure 10:**
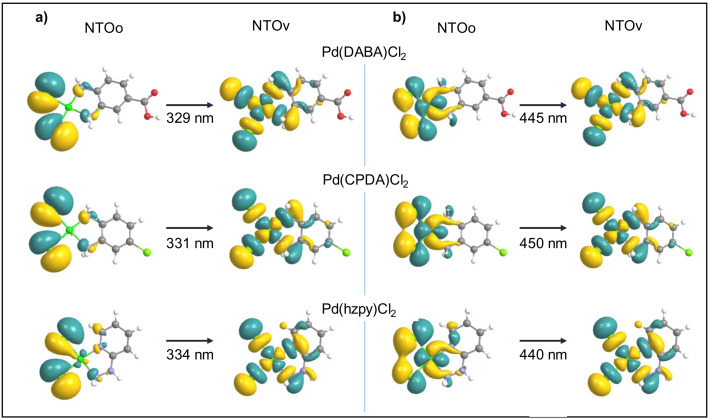
Occupied (NTOo) and virtual (NTOv) Natural Transition Orbitals characterizing the electronic transitions at (**a**) above 300 nm and (**b**) above 400 nm for Pd complexes.

The experimental peaks recorded above 300 nm are quite well reproduced by computations. In particular, weak transitions at 329 nm, 331 nm, and 334 nm are found in the computed spectra for Pd(DABA)Cl_2_, Pd(CPDA)Cl_2_, and Pd(hzpy)Cl_2_, respectively (vs 329 nm, 331 nm, and 334 nm, experimentally determined, Figure S2), and they have a significant LMCT contribution, as suggested by the NTOs analysis reported in Figure 10.

The leaving electron comes from π orbital, whose largest contribution comes from the chloride ligands to a delocalized orbital over the metal center and the ligands. The very weak absorptions around 450 nm can be assigned to metal d–d and LLCT transitions.

**Figure 11:**
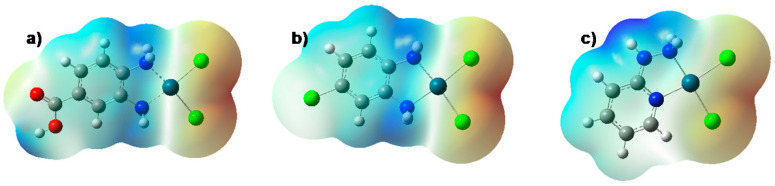
Molecular electrostatic potentials mapping (MEP) for (**a**) for Pd(DABA)Cl_2_, (**b**) Pd(CPCA)Cl_2_, (**c**) Pd(HZPY)Cl_2,_ complexes.

As the analogous Pt(II) compounds, these new complexes display molecular electrostatic potentials (MEP, Figure 11) with the main negative region located on the chloride ions, which make them good leaving groups.

Indeed, it is well known that the mechanism of action of cisplatin-like compounds starts with the activation, by hydrolysis, of the metal complexes generating reactive aquated species able to subsequently interact with DNA, ultimately leading to apoptosis. The chloride ligands’ replacement occurs once the complex enters the cell nucleus, due to the lower chlorine concentration compared with water. The exploration of the chloride–aqua substitution reactions is, therefore, essential to provide useful insights on the kinetics of the process. DFT studies have proven to be accurate in providing both mechanistic details as well as potential energy surfaces for Pt(II) and even Pd(II) compounds [32,33,34,35,36,37,38,39,40,41].

**Figure 12:**
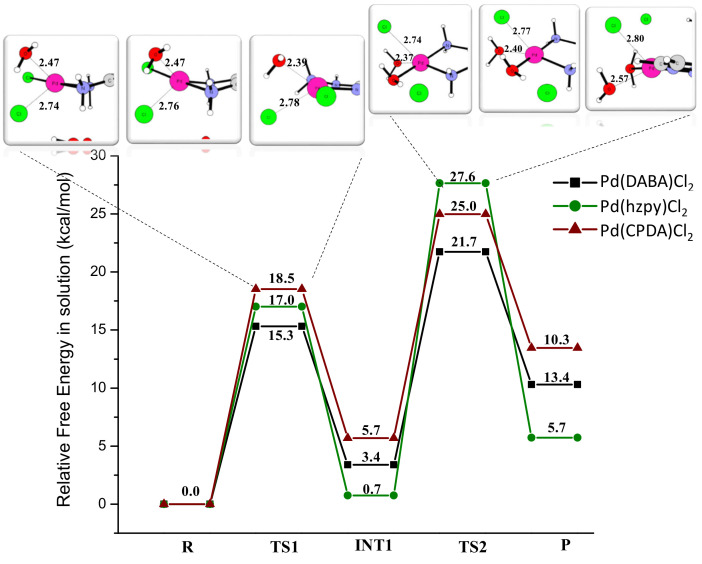
Potential energy profiles for the first and second hydrolysis reaction of the investigated Pd(II) complexes, with snapshots of the first and second transition states (TS1 and TS2), computed in water at the B3LYP/6-31++G(2df,2pd)/SDD-level of theory. The reactants, first intermediate and product of the reaction are referred as R, INT1 and P in the energetic profile, respectively. More details can be found in SI section.

Aiming at providing information on this process, even for our new Pd(II) complexes, the hydrolysis reaction was explored at the DFT-level of theory. The obtained potential energy surface (PES) for all the complexes, together with snapshots of the relevant distances involved in the transition states, are reported in Figure 12. All the stationary points located along the PES are depicted in Figure S6, and their Cartesian coordinates are provided in Table S2. Inspection of the mechanism confirms that the reaction paths follow a second-order nucleophilic substitution (SN2) reaction, with each step of the reaction proceeding via an associative mechanism through a penta-coordinated trigonal bipyramid transition state, in analogy with the established hydrolysis paths of similar compounds.

For each considered complex, the reaction starts with the catalytic water molecule located in the Pd(II) second-coordination sphere (R). The two extra water molecules assist the process, creating a hydrogen-bonding network involving –NH_2_ or –Cl groups of DABA, CPDA, or hzpy ligands. The first transition state (TS1) is characterized by the incoming water molecule at distances comprised between 2.39 Å and 2.47 Å, and leaving the chloride at an average distance of 2.76 Å from the metal center (Figure 12). The latter arranges in a trigonal bipyramidal geometry with water molecules and restores its square-planar geometry in the first aqua-complex (INT1). A second water molecule approaches the Pd(II) center in the second associative transition state (TS2), which represents the rate-determining step (RDS) for all the investigated complexes, ultimately leading to the diaqua complexes (P). From our data emerges that the faster hydrolysis occurs for the Pd(DABA)Cl_2_ complex, with the latter requiring an activation energy to be overcome equal to 15.3 kcal/mol. The first chloride–water substitution requires slightly higher energies for the other two considered complexes; however, this is still very feasible. As previously mentioned, the activation barrier for the second hydrolysis process appears higher, compared to the first one, for all the complexes. Even in the second step, the Pd(DABA)Cl_2_ complex shows the lowest activation barrier, while the rate-determining step is considerably higher in energy for compounds bearing the hzpy bidentate ligand, being the difference between the two significant barriers. Accordingly, results lead us to suggest that our Pd(II) complexes could mainly interact with DNA in their monohydrated form. The small differences between the two computed activation barriers for Pd(DABA)Cl_2_ and Pd(CPDA)Cl_2_, however, also suggest that the formation of diaqua complexes is still a plausible mechanism of action. On the contrary, the high RDS found for Pd(hzpy)Cl_2_ lead us to exclude for it the possibility of reaching the biological target while fully hydrated.

### 2.4. The Antitumor Activities of the Pd Complexes

The antitumor activities of the three palladium complexes were studied in vitro versus three cell lines: breast cancer (MCF-7), prostate carcinoma cell line (PC3) and liver carcinoma cell line (HEPG2). The inhibition and survival percentages are tabulated in (Table 5).

The [Pd(DABA)Cl_2_] complex exhibited the highest inhibition percentage, lying between 68–71%, as reported in Table 5—much closer to that of the reference Vinblastine sulfate, whose measured inhibition % lies between 82–88%. Although smaller, [Pd(DABA)Cl_2_] shows promising activity that deserves further investigation. On the contrary, compared to the other two complexes, [Pd(hzpy)Cl_2_] showed the weakest performance, especially against PC3 and MCF7 cell lines (Table 5). The weak activities of the [Pd(hzpy)Cl_2_] complex could be linked with the higher activation energy found to undergo full hydrolysis, whose possibility cannot be excluded, instead, for the other two compounds, as suggested by the DFT mechanistic studies and free energy profiles. The inhibition values do not have a distinct pattern among the three complexes to enable the structure–activity correlation. Further study may be also conducted in the future to study the cytotoxicity of samples against normal cells.

## 3. Materials and Methods

### 3.1. Materials

All the chemicals and solvents used in the current work were of high purity and were used without further purification. Sodium tetrachloro palladate, 4-chloro–o-phenylenediamine (CPDA), 3,4-diaminobenzoic acid (DABA) and 2-hydraziniopyridine hydrochloride (hzpy) were purchased from Sigma–Aldrich (Saint Louis, MO, USA).

### 3.2. Physical Measurements

The IR spectra of the three complexes were recorded via FT-IR–460 plus (JASCO, Tokyo, Japan). The absorption spectra were recorded using an Optizen spectrophotometer using quartz cell (K Lab, Gunpo-si, Korea). A Varian-Oxford Mercury VX-300 NMR (300 MHz) spectrometer (Agilent, Santa Clara, CA, USA) was used for recording the ^1^H NMR spectra in DMSO-d_6_. Fast atom bombardment mass spectroscopy (FAB-MS) measurements of the Pd(II) complexes were performed via a JMS-AX 500 spectrometer (JEOL, Tokyo, Japan). Thermal analyses of the palladium complexes were performed by using a thermo gravimetric analyzer TGA-50H (Shimadzu, Kyoto, Japan) under a continuous flow of nitrogen (20 mL/min) and heating rate of 10 °C/min, over starting from ambient temperature up to 1000 °C.

### 3.3. Computational Details

All the computations herein presented were performed at DFT- and TDDFT- [42] levels of theory, as implemented in the GAUSSIAN 16 program package. [43] Ground-state optimizations were carried out in water without constraints, by means of the B3LYP exchange–correlation functional (XC) in conjunction with the 6–31+G(d,p) basis and the quasirelativistic Stuttgart–Dresden pseudopotential [44] to describe Pd metal centers. The integral equation formalism polarizable continuum model (IEFPCM) [45,46], setting a dielectric constant equal to ε = 80, was used to simulate the effect of the environment in conjunction with the CPCM polarizable conductor calculation model [47]. Absorption spectra were obtained in water for the singlet ground-state equilibrium structures, using the same basis set and XC functional as for the optimizations. To better describe the kinetic of the hydrolysis process of the Pd(II) compounds, in addition to the implicit solvent, two extra explicit water molecules beyond the catalytic one were added in the system during the exploration of the hydrolysis mechanism. The resulting model was then optimized without constraints during the search of the intermediates and the transition states located along the hydrolysis free energy profiles. Vibrational frequency analysis was carried out at the same level of theory to confirm the nature of all the stationary points. Imaginary frequency values are reported in the SI for each transition state found. The use of implicit solvent and explicit water molecules allow for the proper solvation of the leaving chloride, better accounting for the hydrogen-bonding network during the substitution process. Such a model was previously adopted for the study of similar reactions [36,37,38,39,40,41]. The total free energy in solution, including all non-electrostatic terms (solute–solvent dispersion interaction energy, solute–solvent repulsion interaction energy, and solute cavitation energy), were obtained through single-point calculations with the larger basis set 6–31++G(2df,2pd), and were used to draw the relative free energy profiles of the hydrolysis mechanism. The used protocol allows a direct comparison with results previously obtained for analogous Pt(II) compounds [36,37,38,39,40,41].

### 3.4. Antitumor Activity

The cytotoxicity testing was performed by the assessing of the cellular growth and survival by using a rapid colorimetric assay method at the Tissue Culture Unit at the Regional Centre for Mycology and Biotechnology (RCMB), Al-Azhar University, Cairo, Egypt [48,49]. Three different cancer cell lines were used to investigate the antitumor activities of the palladium complexes, as well as of the reference Vinblastine sulfate, including human breast cancer (MCF-7), prostate carcinoma cell line (PC3), and liver carcinoma cell line (HEPG2). All cell lines were obtained from VACSERA Tissue Culture Unit. The cells were propagated in Dulbecco’s modified Eagle’s medium (DMEM) or RPMI-1640, depending on the type of cell line, supplemented with 10% heat-inactivated fetal bovine serum, 1% L-glutamine, HEPES buffer, and 50 µg/mL gentamycin. All cells were maintained at 37 °C in a humidified atmosphere with 5% CO_2_, and were sub-cultured two times a week during experimentation [48,49]. The concentration of the complexes used in the screening was 100 mg/well. Vinblastine sulfate (100 mg/well) was used by RCMB as a reference, and was treated the same way as the Pd(II) complexes.

### 3.5. Synthesis of the Palladium Complexes

#### 3.5.1. Synthesis of [Pd(DABA)Cl_2_] Complex

The [Pd(DABA)Cl_2_] complex was prepared by mixing Na_2_PdCl_4_ (0.294 g, 1.0 mmol) with DABA ligand (0.152 g, 1.0 mmol) in 20 mL of ethanol. The mixture was adjusted to pH 3.5 and stirred for 4 h. The solid of each complex was filtered off and washed thoroughly with ethanol, followed by diethyl ether; the solid complex was dried under vacuum and subjected to analysis. Yield: (0.237 g, 72%). Found: C, 25.35; H, 2.23; N, 7.56. Anal. Calc. for C_7_H_8_N_2_O_2_PdCl_2_: C, 25.52; H, 2.45; N, 8.50. FT-IR (KBr, cm^−1^) υ: 3213 (s), 1720 (s), 1272 (m), 1141 (s), 771 (s), and 428 (m). ^1^H-NMR (δ, ppm) (DMSO-d_6_): aromatic protons of DABA (7.69–8.33, 3H), NH_2_ groups (3.49 ppm, s, 2H). MS (FAB-MS): *m*/*z* = 328 (M^+^-H). UV–Vis: 315, 330, 340, 380, 690, and 715 nm. UV–Vis spectra are reported in Figure S1.

#### 3.5.2. Synthesis of [Pd(CPDA)Cl_2_] Complex

The [Pd(CPDA)Cl_2_] complex was prepared by mixing Na_2_PdCl_4_ (0.294 g, 1.0 mmol) with CPDA ligand (0.142 g, 1.0 mmol) in 20 mL of ethanol. The mixture was adjusted to pH 3.5 and stirred for 4 h. The solid of each complex was filtered off and washed thoroughly with ethanol, followed by diethyl ether; the solid complex was dried under vacuum and subjected to analysis. Yield: 0.236 g (74%). Found: C, 21.89; H, 2.54; N, 7.98. Anal. Calc. for C_6_H_7_N_2_PdCl_3_: C, 22.53; H, 2.21; N, 8.76. FT-IR (KBr, cm^−1^) υ: 3213 (s), 3243 (s), 1265 (m), 1145 (s), 779 (s), and 428 (m). ^1^H-NMR (δ, ppm) (DMSO-d_6_): aromatic protons of CPDA ligand (7.69–8.33, 3H), NH_2_ groups (4.79 ppm, s, 2H). MS (FAB-MS): *m*/*z* = 320 (M^+^). UV–Vis: 315, 365, 380, 560, 685, and 715 nm.

#### 3.5.3. Synthesis of [Pd(hzpy)Cl_2_] Complex

The [Pd(hzpy)Cl_2_] complex was prepared by mixing Na_2_PdCl_4_ (0.294 g, 1.0 mmol) with hzpy ligand (0.182 g, 1.0 mmol) in 20 mL of ethanol. The mixture was adjusted to pH 3.5 and stirred for 4 h. The solid of each complex was filtered off and washed thoroughly with ethanol, followed by diethyl ether; the solid complex was dried under vacuum and subjected to analysis. Yield: (0.21 g, 73%). Found: C, 20.32; H, 2.67; N, 13.58. Anal. Calc. for C_5_H_7_N_3_PdCl_2_: C, 20.96; H, 2.46; N, 14.67. FT-IR (KBr, cm^−1^) υ: 3274 (s), 3159 (s), 1284 (m), 1188 (s), 763 (s), and 466 (m). ^1^H-NMR (δ, ppm) (DMSO-d_6_): aromatic protons of hzpy ligand (7.69–8.33, 3H), NH and NH_2_ group (6.69, m, 3H). MS (FAB-MS): *m*/*z* = 286 (M^+^). UV–Vis: 310, 370, and 515 nm.

## 4. Conclusions

A joint experimental and theoretical study was carried out on three novel palladium complexes with potential activities as anticancer agents. The compounds were synthesized, spectroscopically and thermally characterized, their hydrolysis mechanism as well as their photophysical properties were explored by means of DFT and TDDFT calculations, and their cytotoxic effects were tested against breast cancer (MCF-7), prostate carcinoma cell line (PC3) and liver carcinoma cell line (HEPG2). The [Pd(DABA)Cl_2_] complex exhibited the highest inhibition percentage, lying between 68–71%. In addition to elucidating the steps of the SN_2_ mechanism employed by these complexes to hydrolyze, the outcomes of the DFT exploration also indicate a faster chloride–water substitution in the case of the [Pd(DABA)Cl_2_] complex, that could be linked with the superior performances of that compound. For all the complexes, the rate determining step is represented by the second hydrolysis process. In any case, while the significant higher activation energy found for the second chloride–water substitution mechanism computed for Pd(hzpy)Cl_2_ leads to exclude the possibility for such complex to undergo full hydrolysis, the same possibility appears more plausible for the other two compounds for which the difference between the two activation energies is smaller and for which the formation of diaqua complexes could be still a feasible process.

## Figures and Tables

**Table 1:** Experimental vibrational frequencies of the Pd complexes.

Complex	Obs	Assignment	Complex	Obs	Assignment	Complex	Obs	Assignment
[Pd(DABA)Cl_2_]	3213	υ(NH_2_)	[Pd(CPDA)Cl_2_]	3213	υ(NH_2_)	[Pd(hzpy)Cl_2_]	3274	υ(NH_2_)
	1720	υ(C=O)		3143	υ(NH_2_)		3159	υ(NH_2_)
	1242	(ρt NH_2_)		1265	(ρt NH_2_)		1284	(ρt NH_2_)
	1172	(ρwNH_2_)		1145	(ρwNH_2_)		1188	(ρwNH_2_)
	771	(ρrNH_2_)		779	(ρrNH_2_)		763	(ρrNH_2_)
	428	υ(M-N)		428	υ(M-N)		466	υ(M-N)

**Table 2:** Important mass peaks of palladium complexes.

Complex	Molar Mass	*m*/*z* Values
[Pd(DABA)Cl_2_]	329.48	328, 192, 150, 147, 109, 107, 106
[Pd(CPDA)Cl_2_]	319.91	320, 180, 141,110, 109, 106
[Pd(hzpy)Cl_2_]	286.46	286, 142, 109, 110, 109, 106, 104

**Table 3:** Thermo-analytical data of palladium complexes.

Complexes	TG Range (K)	DTA_max_ (K)	Mass Loss Found (calc. %)	Assignment of the Removed Species	Metallic Residue Found (calc.%)
[Pd(DABA)Cl_2_]	477–642	631	59.8, (57.1)	C_7_H_8_N_2_O_2_, 1/2Cl_2_	Pd
	708–1201	751	9.0, (10.7)	1/2Cl_2_	31.2, (32.5)
[Pd(CPDA)Cl_2_]	550–682	572	55.21, (55.6)	(C_6_H_7_N_2_Cl), 1/2Cl_2_	Pd
	1057–1224	1056	12.74, (11.76)	1/2 Cl_2_	33.06, (33.28)
[Pd(hzpy)Cl_2_]	483–618	488	51.2, (53.9)	C_5_H_7_N_3_+1/2Cl_2_	Pd
	620–1179	1141	10.6, (13.2)	Cl	38.2, (38.4)

**Table 4:** The thermodynamic parameters of the thermal decomposition of the palladium complex.

Complex	Decomposition Temperature (K)	∆E/ KJ mol^−1^	R^2^	∆S/ J K^−1^ mol^−1^	∆H/ KJ mol^−1^	∆G/ KJ mol^−1^
[Pd(DABA)Cl_2_]	477–642 708–1201	55 62	0.74 0.62	−170 −228	−51 −52	140 313
		107		−398	103	453
[Pd(CPDA)Cl_2_]	550–682 1057–1224	217 356	0.98 0.98	76 38.5	211 346	162 303
		573		114.5	557	465
[Pd(hzpy)Cl_2_]	483–617 620–1179	43 537	0.86 0.98	−177 599	39 531	128 153
		580		422	570	281

**Table 5:** Cytotoxicity activity of the palladium complexes.

Complexes	PC3	MCF7	HEPG2
	Inhibition%	Inhibition%	Inhibition%
[Pd(DABA)]Cl_2_	71	70	68
[Pd(CPDA)Cl_2_]	56	55	53
[Pd(hzpy)Cl_2_]	44	49	62
Vinblastine Sulfate	82	81	88

## Data Availability

Not applicable.

## Supplementary Materials

The following are available online, Figure S1: UV–Vis spectra; Figure S2: Spectral data of the N,N diamino ligands; Figure S3: HOMO-1, HOMO, LUMO, LUMO+1 MOs plots with the indication of the main orbital composition (%) and IR spectra; Figure S4: Computed Spectra in DMF at the B3LYP/6-31+G(d,p)/SDD-level of theory, for all the Pd complexes; Figure S5: Occupied (NTOo) and virtual (NTOv) Natural Transition Orbitals of the main electronic transitions quoted as A1–A4, as reported in Figure S4; Figure S6: Optimized structures for the first and second hydrolysis processes; Table S1: Selected bond lengths and angles; Table S2: Cartesian coordinates of all the intermediates and transition states located along the hydrolysis free energy profiles.

## Sample Availability

Samples of the compounds are available from the authors.
