# Supplementary material for: Synthesis, Characterization, Thermal Analysis, DFT, and Cytotoxicity of Palladium Complexes with Nitrogen-Donor Ligands
Source: Molecules. 2022 Jan 31;27(3):964. doi: 10.3390/molecules27030964 (PMC8837960; doi:10.3390/molecules27030964)
Supplement: Supplementary file 1 [file molecules-27-00964-s001.zip › molecules-1551373-supplementary.pdf]

Supplementary materials

-Contents-

Figure S1: UV-Vis spectra of a) [Pd(DABA)Cl_2]; b) [Pd(CPDA)Cl_2]; c) [Pd(hzpy)Cl_2] complexes

Figure S2: Spectral data of the N,N diamino ligands and IR spectra of a) 3,4- diaminobenzoic acid (DABA); b) 4- chloro-o-phenylendiamino (CPDA); c) 2-hydrazinopyridine (hzpy)

Figure S3: HOMO-1, HOMO, LUMO, LUMO+1 MOs plots with the indication of the main orbital composition (%), for Pd(DABA)Cl_2 , Pd(CPCA)Cl_2 ,Pd(HZPY)$Cl_{2,}$ in the singlet ground state;

Figure S4: Computed Spectra in DMF at B3LYP/6-31+G(d,p)/SDD level of theory, for all the Pd-complexes

Figure S5: Occupied (NTOo) and virtual (NTOv) Natural Transition Orbitals of the main electronic transitions quoted as A1-A4 as reported in Figure S4

Figure S6: Optimized structures for the first and second hydrolysis processes of a) [Pd(DABA)Cl_2]; b) [Pd(CPDA)Cl_2]; c) [Pd(hzpy)Cl_2] complexes, in water phase, at B3LYP/ 6-31+G(d,p)/SDD level of theory

Table S1: Selected bond lengths and angles for a) [Pd(DABA)Cl_2]; b) [Pd(CPDA)Cl_2]; c) [Pd(hzpy)Cl_2] optimized complexes

Table S2: Cartesian coordinates of all the intermediates and transition states located along the hydrolysis free energy profiles, optimized in water phase at B3LYP/ 6-31+G(d,p)/SDD level of theory

-Figure S1-

UV-Vis spectra recorded in DMF of a) [Pd(DABA)Cl_2]; b) [Pd(CPDA)Cl_2]; c) [Pd(hzpy)Cl_2] complexes

a)

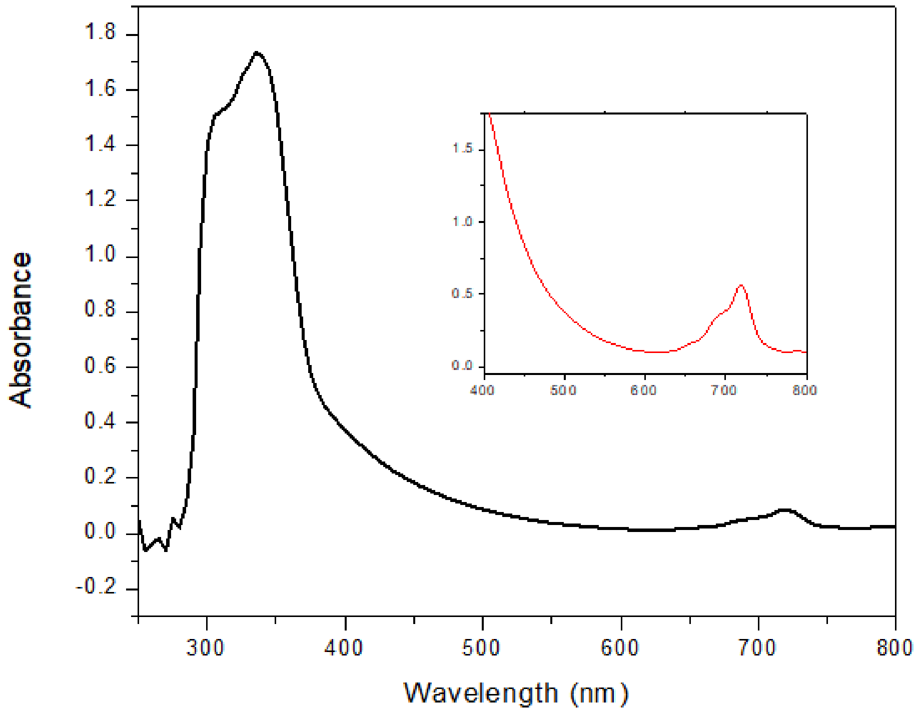

b)

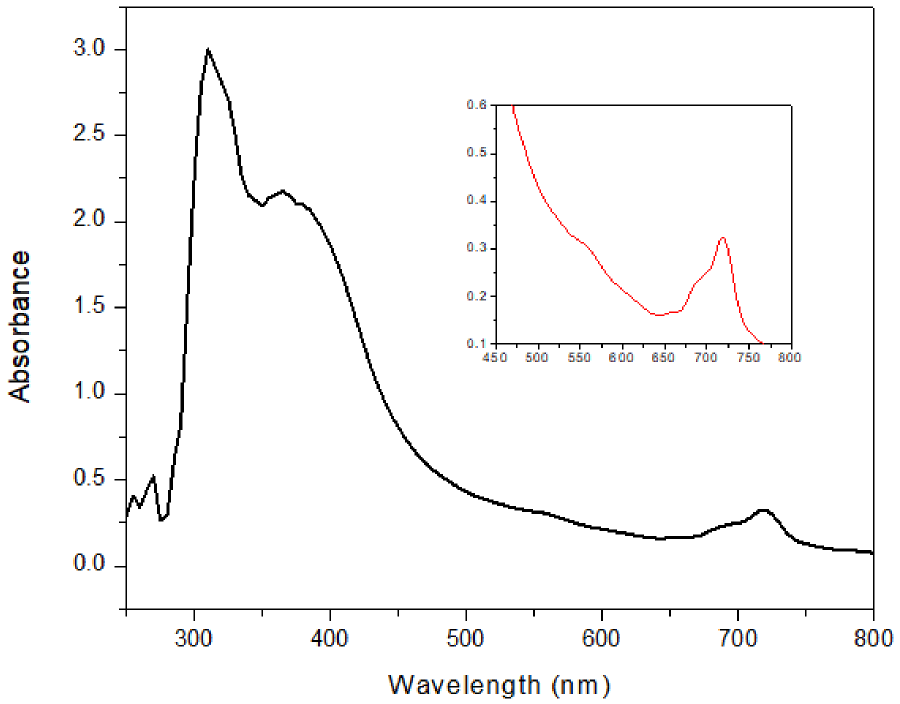

c)

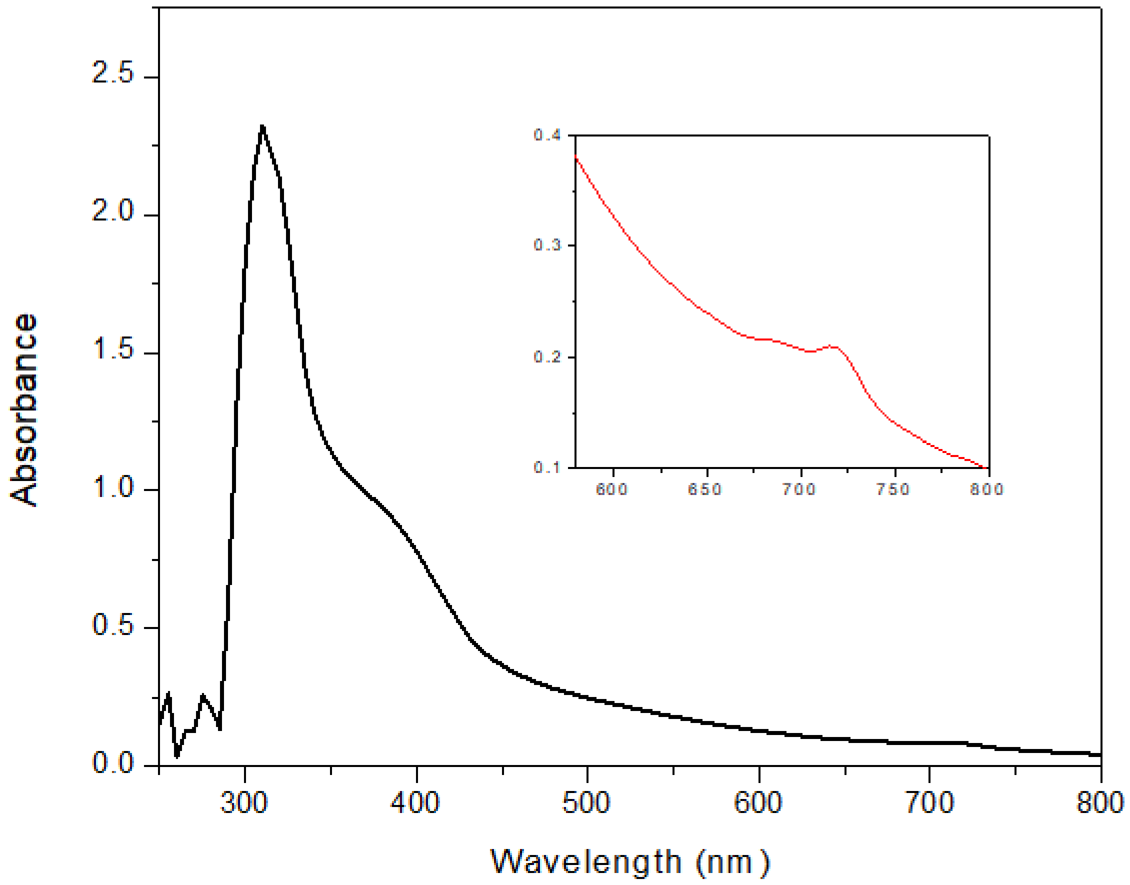

Absorbance
Wavelength (nm)
0.0
0.5
1.0
1.5
2.0
2.5
300
400
500
600
700
800
0.1
0.2
0.3
0.4
600
650
700
750
800

-Figure S2-

Spectral data of the N,N diamino ligands and IR spectra of a) 3,4- diaminobenzoic acid (DABA); b) 4- chloro-o-phenylendiamino (CPDA); c) 2-hydrazinopyridine (hzpy)

a)

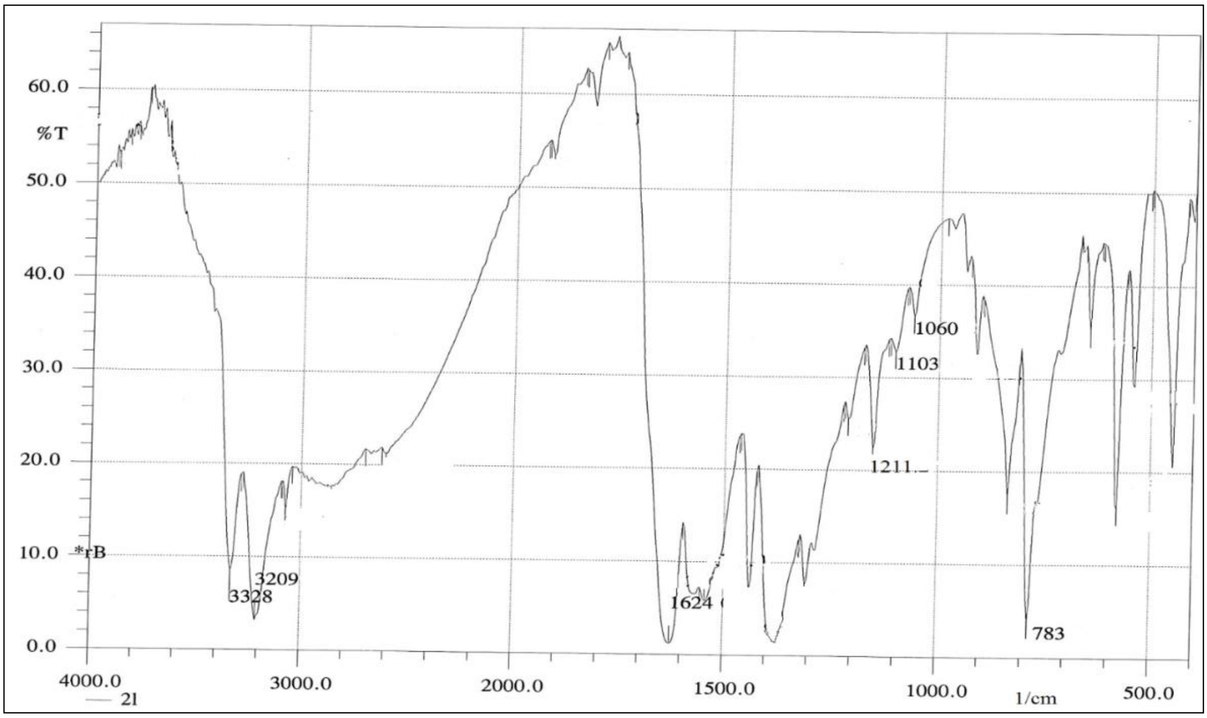

1.1.3,4- diaminobenzoic acid (DABA)

The IR spectrum of the 3,4-diaminobenzoic acid (DABA) ligand; **Fig. S1a**, showed a characteristic band at 3328 cm^{-1} and 3205 cm^{-1} which were assigned to the stretching vibration of the NH_2 group. The deformation vibration of the NH_2 group were observed at 1211cm^{-1} (ρt NH_2), (ρw NH_2), 1153 cm^{-1} and 783 cm^{-1} (ρr NH_2). The spectrum showed also a strong peak at 1624 cm^{-1} which was assigned to carbonyl group vibration (υC=O) effected by hydrogen bonding.

b)

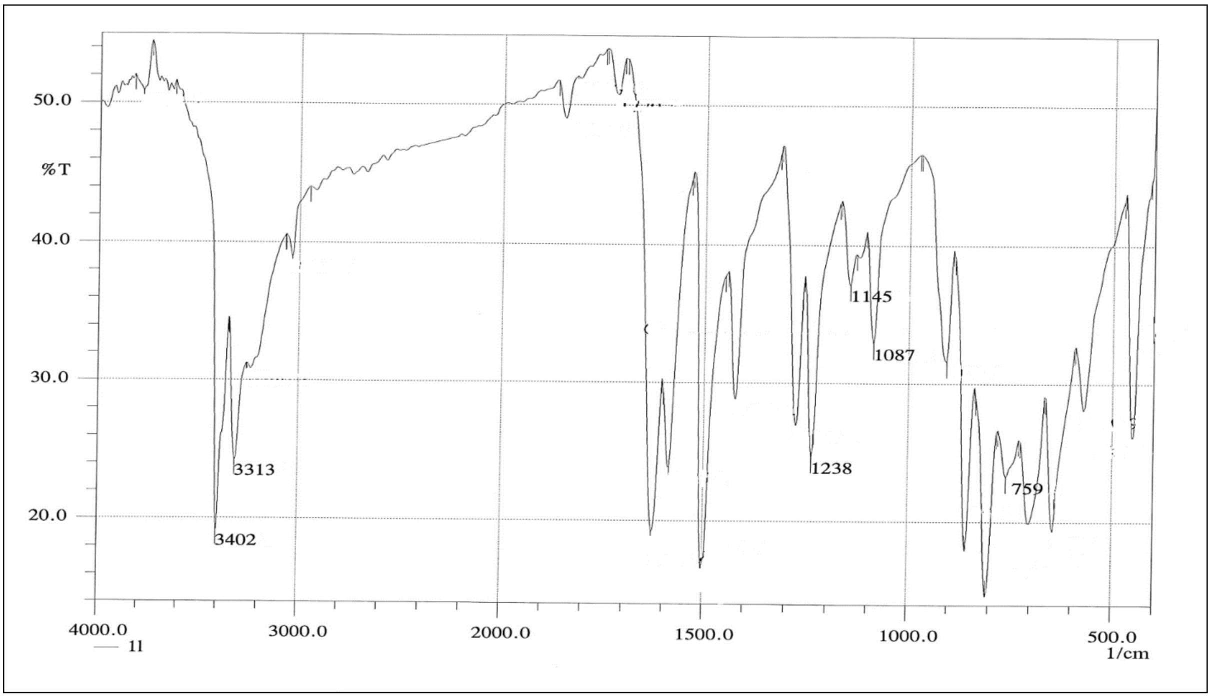

1.2.4-chloro-o-phenlyendiamino (CPDA)

The IR spectrum of the 4-chloro-o-phenlyendiamino (CPDA) ligand; **Fig. S1b**, showed a characteristic band at 3402 cm^{-1} and 3313 cm^{-1} which were assigned to the stretching vibration of the NH_2 group. The deformation vibration of the NH_2 group were observed at 1276 cm^{-1} (ρt NH_2), (ρw NH_2), 1145 cm^{-1} and 795 cm^{-1} ($\rho r NH_2$).

c)

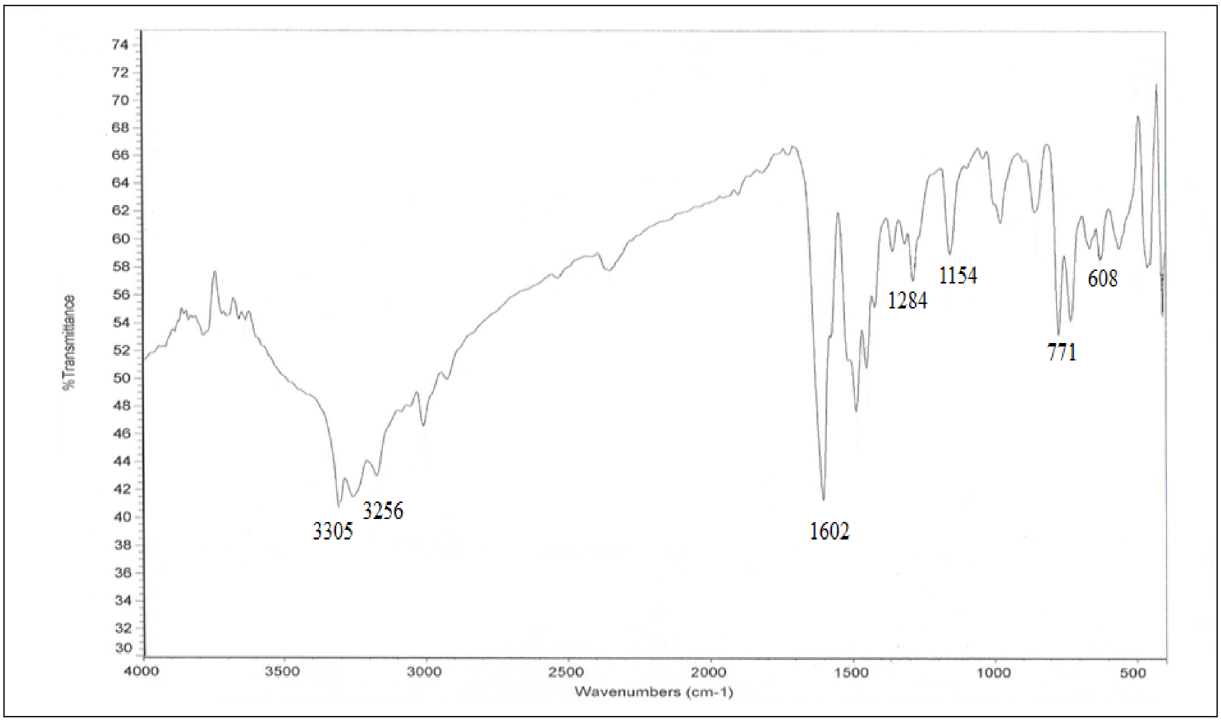

2-hydrizinopyridin (hzpy)

The IR spectrum of the 2- hydrizinopyridine (hzpy) ligand; **Fig. S1c**, showed a characteristic band at 3305 and 3256 cm^{-1} which were assigned to the stretching vibration of the NH_2 group. The deformation vibration of the NH_2 group were observed at 1284 cm^{-1} (ρt NH_2;), (ρw NH_2;), 1154 cm^{-1} and 771 cm^{-1} (ρrNH_2;). The peak at 1602 cm^{-1} may by assigned to the (H–C=N) of the (py) ring. The pyridyl vibration (py) vibrations were at 608 cm^{-1}(in-plane ring deformation) and at 406 cm^{-1} (out-of-plane ring deformation).

-Figure S3-

HOMO-1, HOMO, LUMO, LUMO+1 MOs plots with the indication of the main orbital composition (%), for Pd(DABA)Cl_2 , Pd(CPCA)Cl_2 ,Pd(HZPY)Cl_2, in the singlet ground state

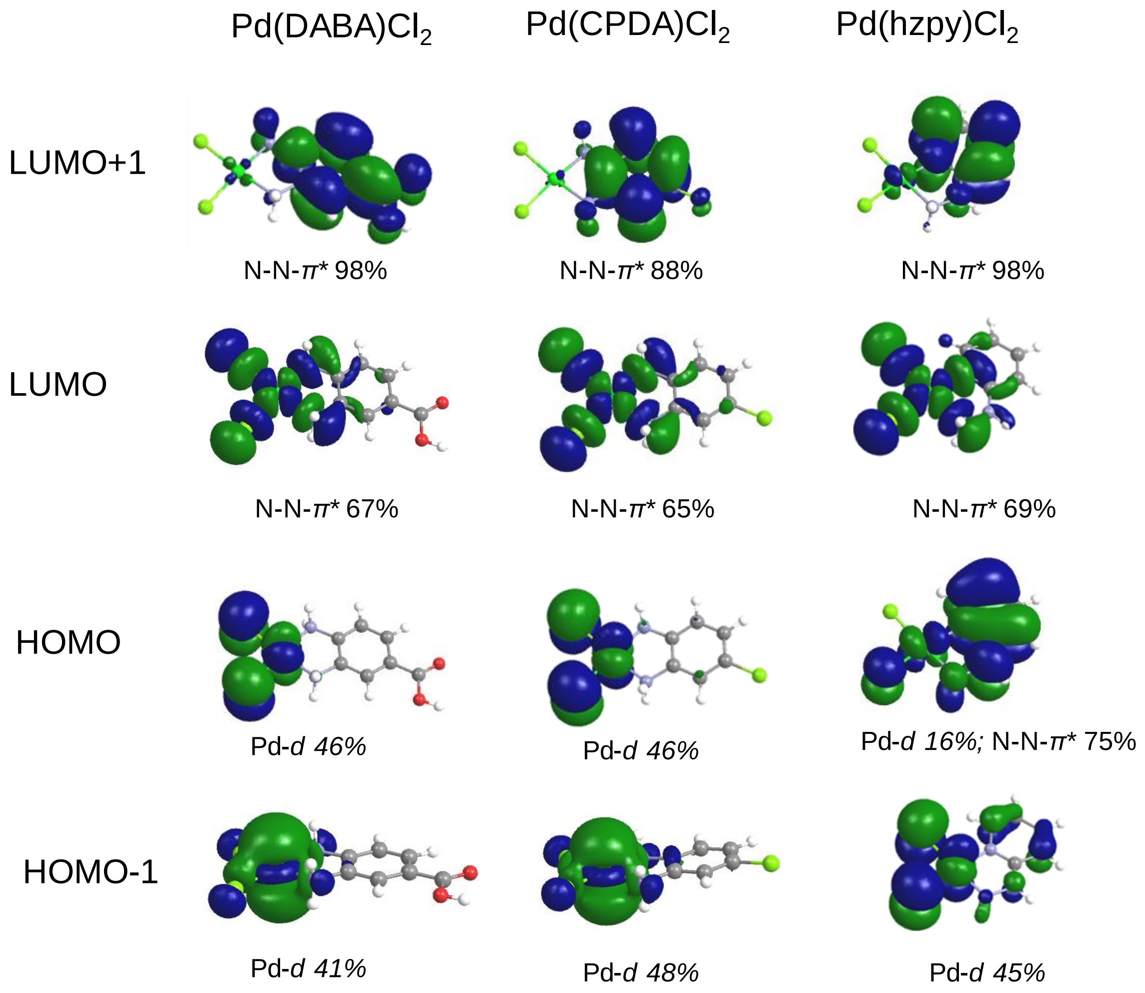

-Figure S4-

Computed Spectra in DMF at B3LYP/6-31+G*/SDD level of theory, for all the Pd-complexes

$Pd(DABA)Cl_2$

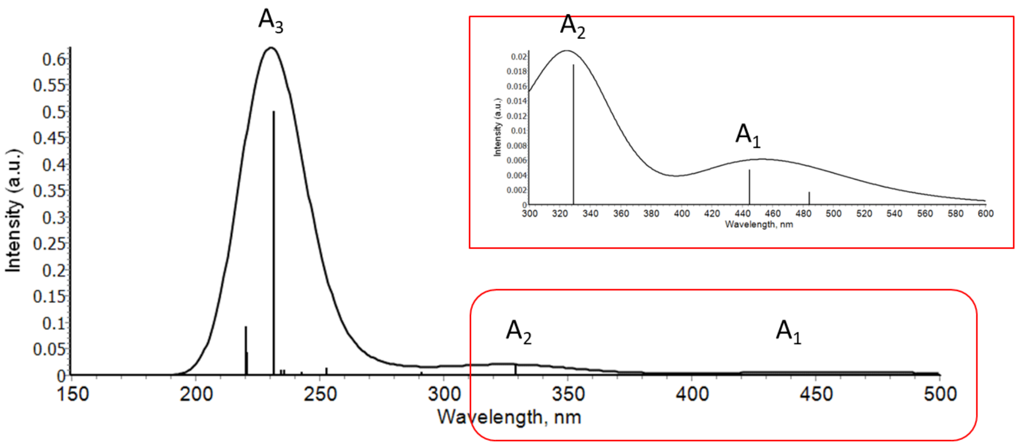

$Pd(CPDA)Cl_2$

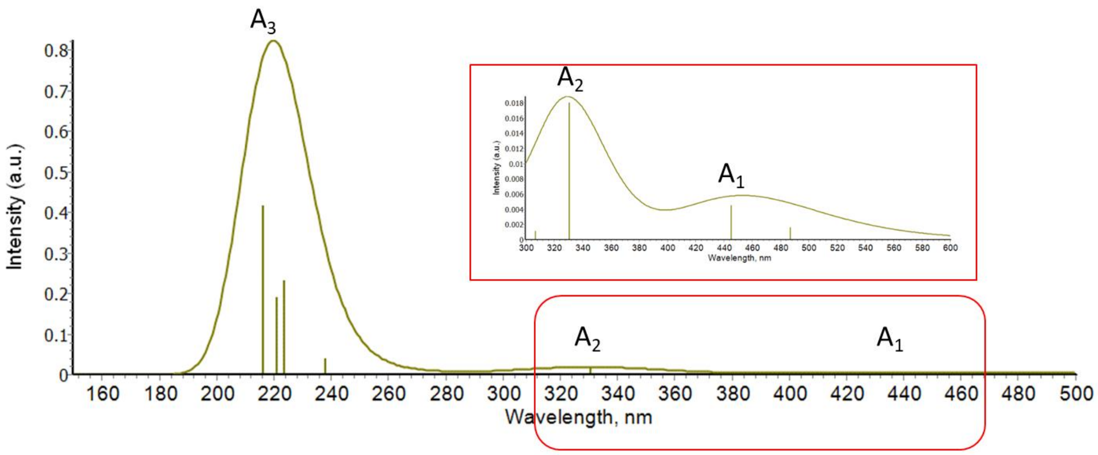

$Pd(hzpy)Cl_2$

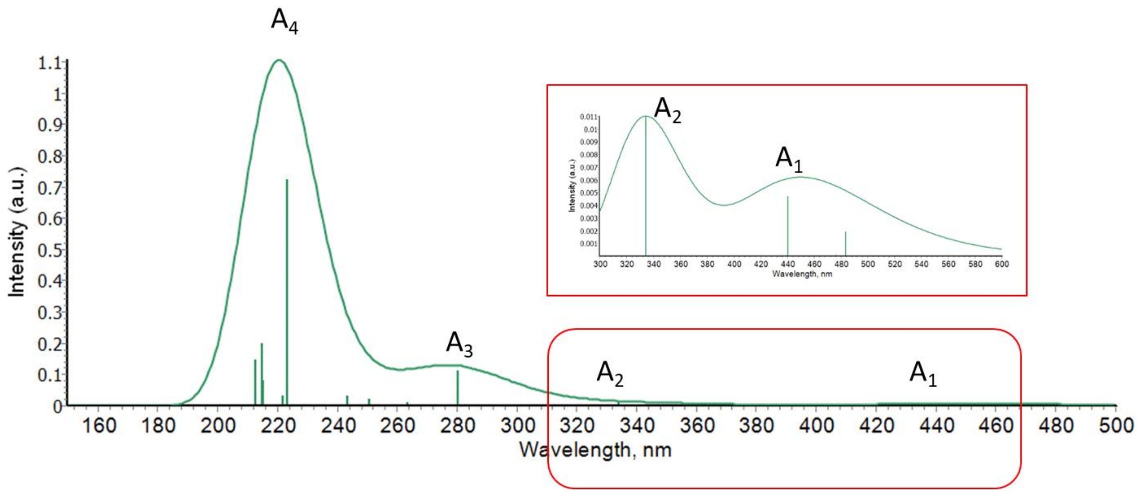

-Figure S5-

Occupied (NTOo) and virtual (NTOv) Natural Transition Orbitals of the main electronic transitions quoted as A1-A4 as reported in Figure S4

a) Pd(DABA)Cl_2

NTOo | **NTOv**

A1
445 nm

A2
329 nm

A3
232 nm

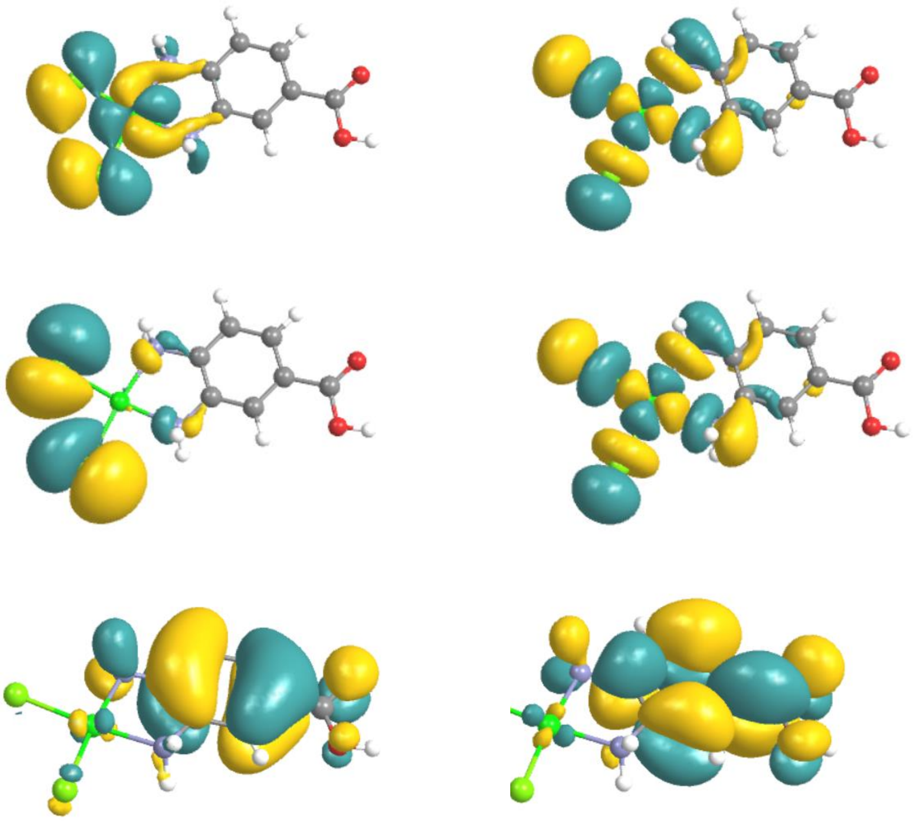

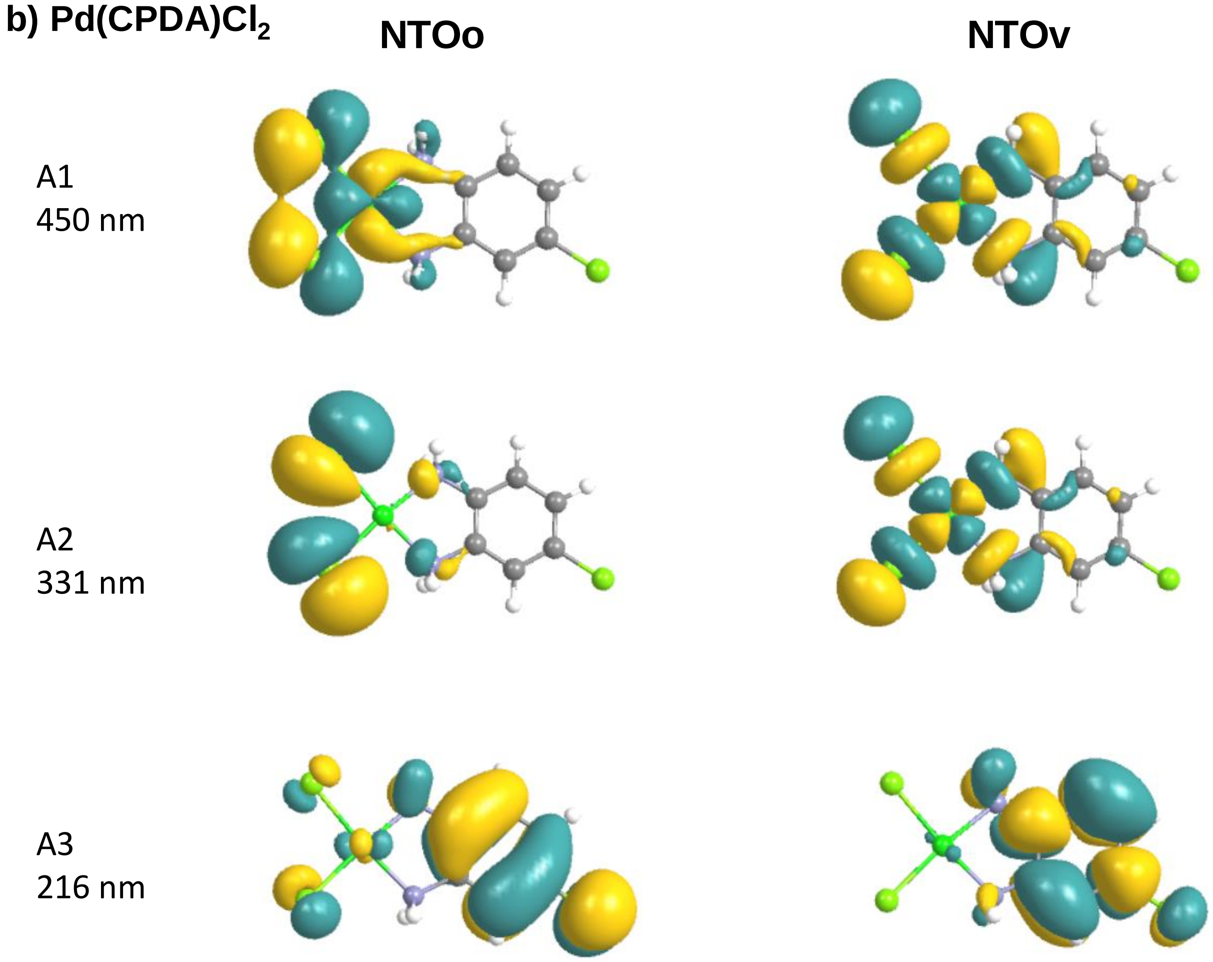
b) Pd(CPDA)Cl_2
NTOo
NTOv
A1
450 nm
A2
331 nm
A3
216 nm

c) Pd(hzpy)Cl_2 NTOo NTOv

A1 440 nm

A2 334 nm

A3 280 nm

A4 223 nm

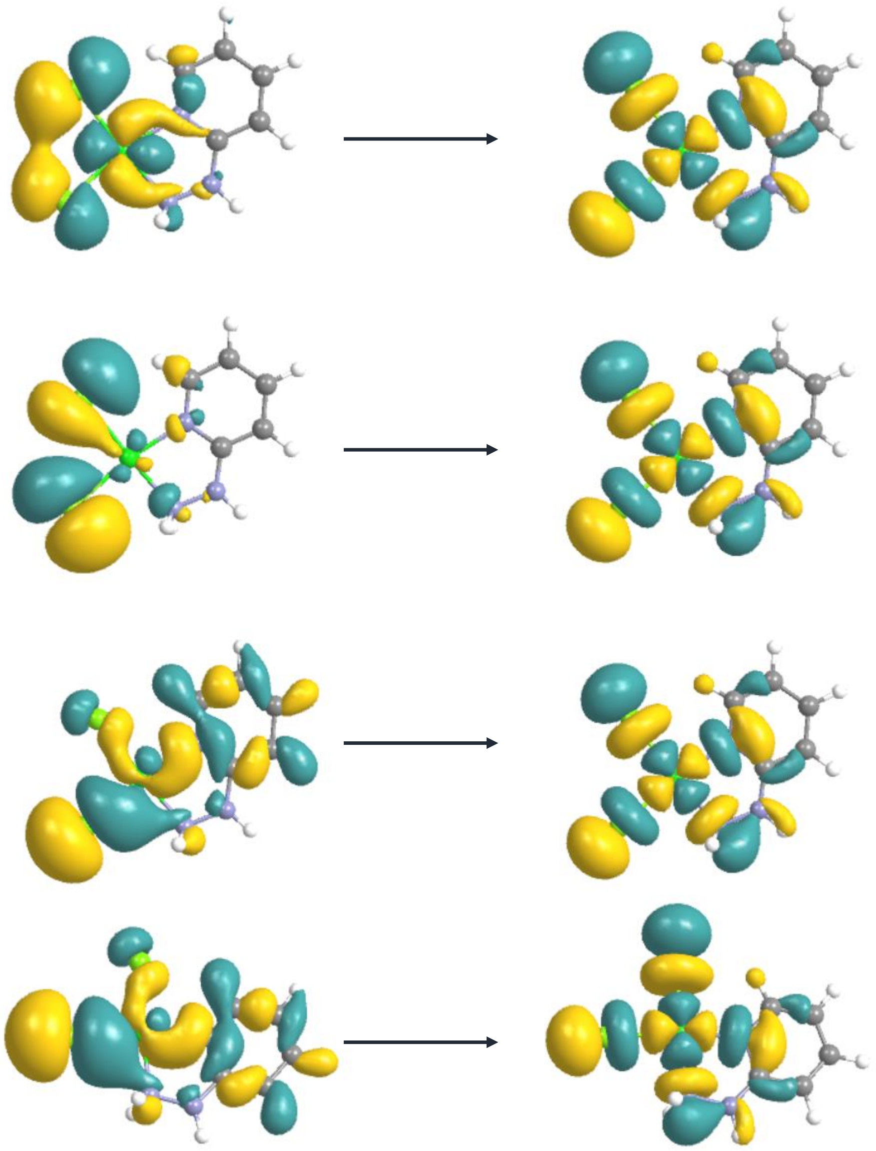

-Figure S6-

Optimized structures for the first and second hydrolysis processes of a) [Pd(DABA)Cl_2]; b) [Pd(CPDA)Cl_2]; c) [Pd(hzpy)Cl_2] complexes, in water phase, at B3LYP/ 6-31+G(d,p)/SDD level of theory

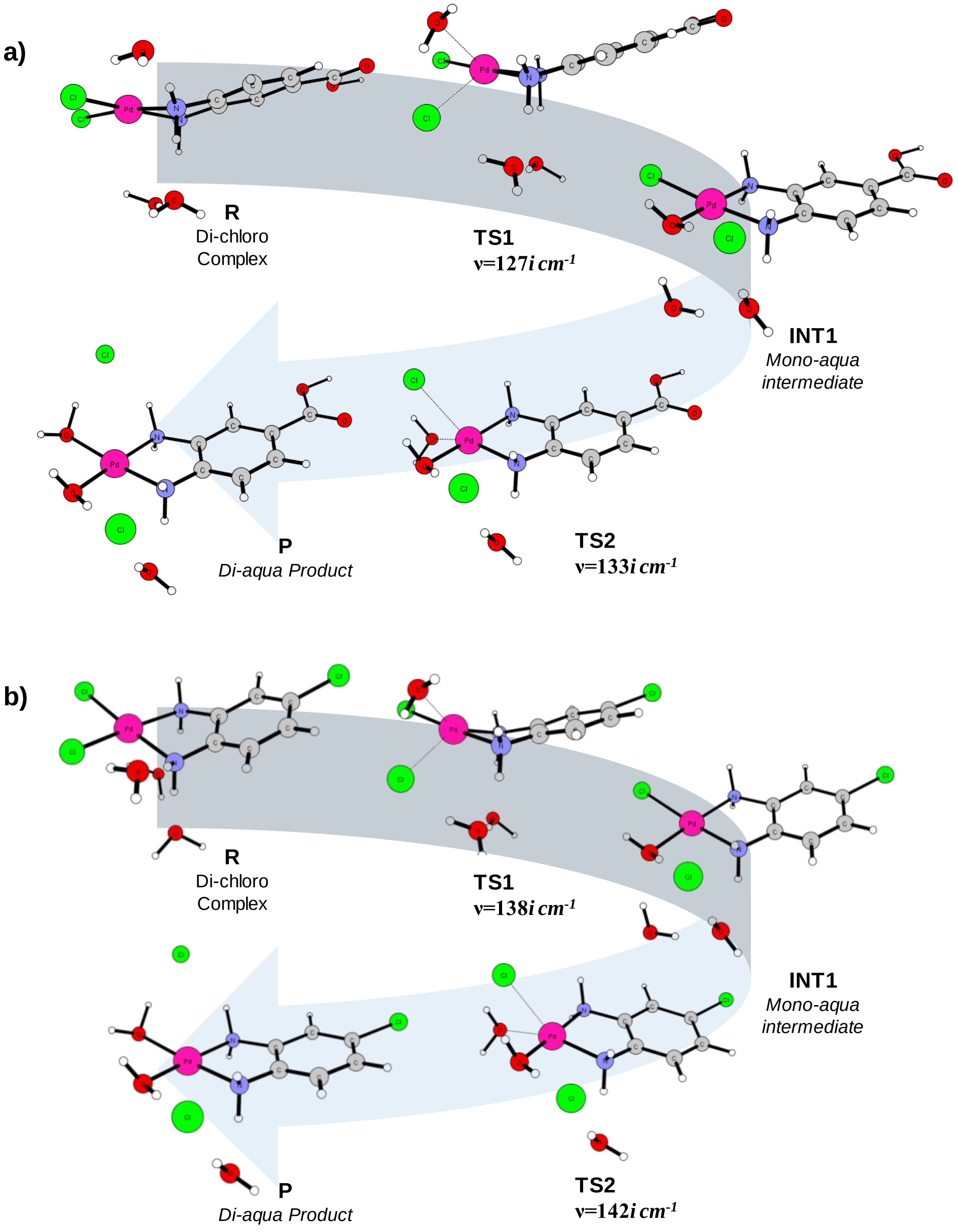

c)

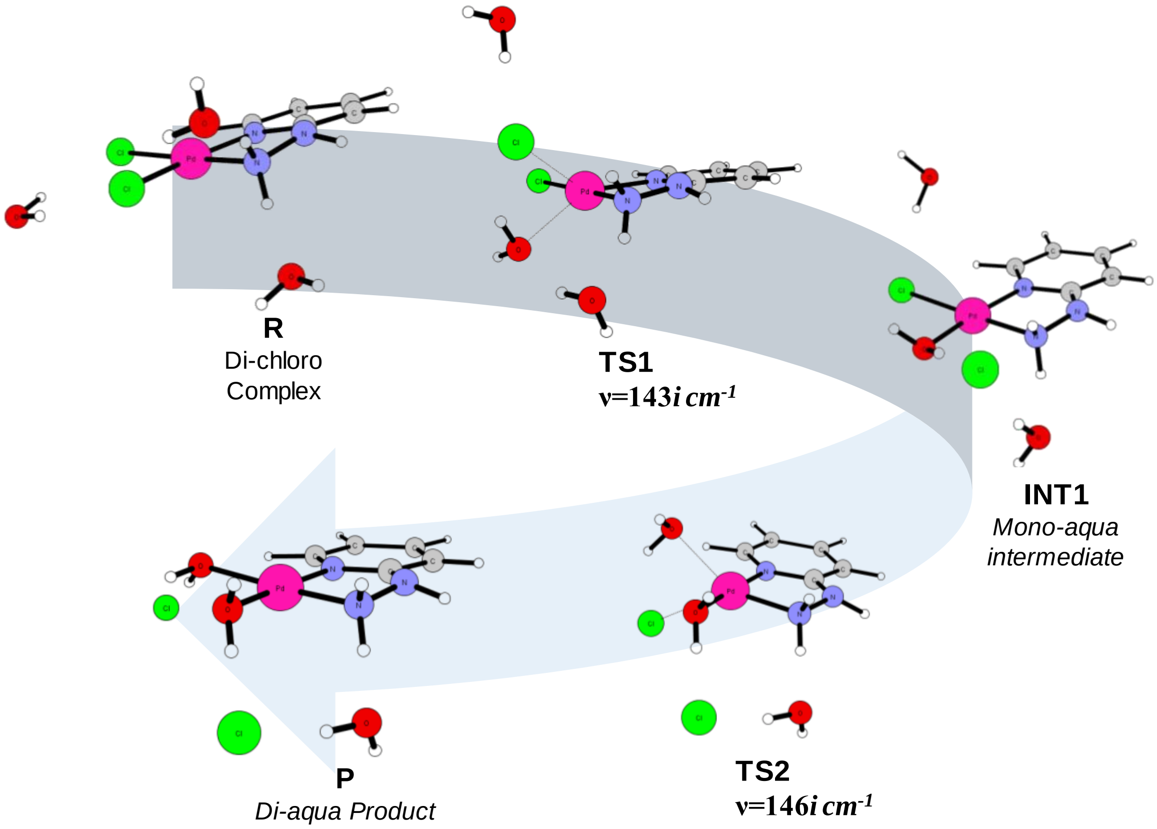
R
Di-chloro
Complex
TS1
ν=143i cm-1
INT1
Mono-aqua
intermediate
TS2
ν=146i cm-1
P
Di-aqua Product

-Table S1-

Selected bond lengths and angles for a) [Pd(DABA)Cl_2]; b) [Pd(CPDA)Cl_2]; c) [Pd(hzpy)Cl_2] optimized complexes and d) structures with numbering

a)

Bond length	Å	NBO	Bond angles	degree
Pd-N1	2.08	Pd 0.34	N1-Pd-Cl1	90.80
Pd-N2	2.08	Cl1 -0.49	N1-Pd-Cl2	174.21
Pd-Cl1	2.34	Cl2 -0.49	N1-Pd-N2	83.08
Pd-Cl2	2.34	N1 -0.82	N2-Pd-Cl1	173.87
		N1 -0.81	N2-Pd-Cl2	91.14
			Cl1-Pd-Cl2	94.99

b)

Bond length	Å	NBO	Bond angles	degree
Pd-N1	2.08	Pd0.34	N1Pd-Cl1	91.02
Pd-N2	2.08	N1-0.81	N1Pd-Cl2	174.39
Pd-Cl1	2.35	N2 -0.81	N1-Pd-N2	83.13
PdCl2	2.35	Cl1-0.49	N2-Pd-Cl1	174.14
		Cl2-0.49	N2-Pd-Cl2	91.27
			Cl1-Pd-Cl2	94.59

c)

Bond length	Å	NBO	Bond angles	degree
Pd-N1	2.05	Pd 0.39	N1-Pd-Cl1	95.44
Pd-N2	2.07	N1 -0.61	N1-Pd-Cl2	171.16
Pd-Cl1	2.34	N2 -0.52	N1-Pd-N2	81.03
Pd-Cl2	2.35	Cl1 -0.49	N2-Pd-Cl1	176.47
		Cl2 -0.48	N2-Pd-Cl2	90.13
			Cl1-Pd-Cl2	93.40

d)

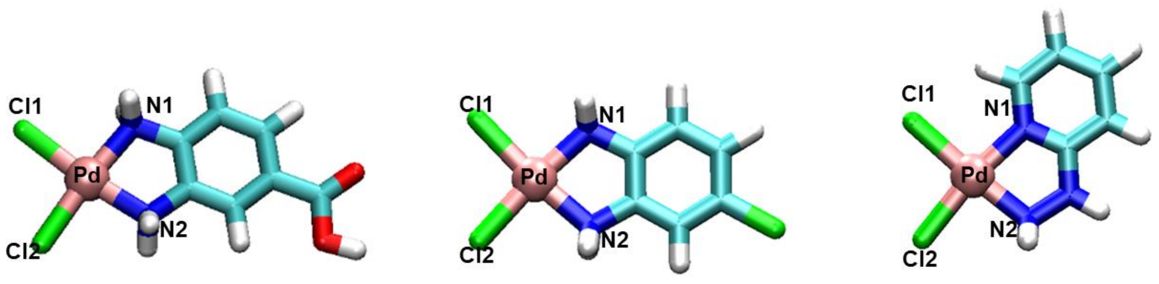

Pd(DABA)Cl_2 Pd(CPDA)Cl_2 Pd(HZPY)Cl_2

Table S2-

Cartesian coordinates of all the intermediates and transition states located along the hydrolysis free energy profiles for a) [Pd(DABA)Cl_2]; b) [Pd(CPDA)Cl_2]; c) [Pd(hzpy)Cl_2]

a) [Pd(DABA)Cl_2];

R

C	0.806340	-0.127653	0.268758
C	0.703115	1.187407	-0.193405
C	1.845901	1.890557	-0.571490
C	3.090317	1.275179	-0.469727
C	3.196321	-0.032720	0.016229
C	2.045858	-0.741835	0.386557
H	1.757558	2.904303	-0.943010
H	3.993275	1.796996	-0.760556
H	2.122434	-1.759846	0.746526
N	-0.625466	1.763764	-0.252877
H	-0.823032	2.259688	0.631027
H	-0.750521	2.410531	-1.055448
N	-0.436675	-0.806239	0.592672
H	-0.434123	-1.774384	0.277992
H	-0.649929	-0.792815	1.611720
C	4.552846	-0.628202	0.138985
O	4.529575	-1.888320	0.614082
H	5.450137	-2.193203	0.671435
O	5.584907	-0.045696	-0.140518
Pd	-2.036239	0.237206	-0.212055
Cl	-3.741726	1.569535	-1.162318
Cl	-3.444316	-1.617188	0.154813
O	-1.420274	3.227004	-2.520391
H	-1.540424	4.176043	-2.387141
H	-2.296087	2.838941	-2.329217
O	-1.676751	-0.377180	3.063717
H	-1.624222	0.596317	2.959606
H	-2.501839	-0.627304	2.620704
O	-1.228296	2.357772	2.527260
H	-0.499399	2.696706	3.064589
H	-1.967893	2.953285	2.709136

TS1

C	1.389568	-0.368045	-0.354905
C	1.140680	0.992654	-0.161027
C	2.177822	1.918997	-0.209553
C	3.471705	1.475167	-0.471451
C	3.724149	0.114815	-0.678251
C	2.677986	-0.816194	-0.615325
H	1.971178	2.972730	-0.060879
H	4.295130	2.175294	-0.528150
H	2.871140	-1.869401	-0.777221
N	-0.242965	1.377436	0.073905
H	-0.431429	1.485828	1.087021
H	-0.454323	2.262135	-0.384725
N	0.249275	-1.260386	-0.254520
H	0.316058	-2.050296	-0.892997
H	0.142424	-1.604468	0.730987

C	5.123666	-0.304673	-0.961553
O	5.237005	-1.624675	-1.200975
H	6.176617	-1.805071	-1.368179
O	6.076168	0.453175	-0.979318
Pd	-1.484526	-0.166183	-0.552105
Cl	-3.566466	0.976728	0.825238
Cl	-2.660016	-2.056302	-1.251055
O	-2.806388	1.275260	-2.062106
H	-2.330800	2.079375	-2.310431
H	-3.349464	1.522690	-1.287741
O	-0.249633	-1.706720	2.418459
H	0.500808	-1.901393	2.992814
H	-0.549403	-0.803066	2.668466
O	-1.118233	0.873153	2.716494
H	-1.150006	1.380749	3.537458
H	-2.027188	0.887566	2.340298

INT1

C	1.329178	-0.539127	0.155048
C	1.069515	0.789800	0.483634
C	2.100019	1.730535	0.467719
C	3.385459	1.326140	0.127911
C	3.646281	-0.012120	-0.192111
C	2.613286	-0.954688	-0.176972
H	1.889398	2.762953	0.720419
H	4.203273	2.035439	0.104352
H	2.811696	-1.989711	-0.425527
N	-0.291087	1.144379	0.844380
H	-0.358499	1.477994	1.825781
H	-0.643880	1.930647	0.287042
N	0.199735	-1.468076	0.169735
H	0.100142	-1.943009	-0.728327
H	0.333100	-2.198884	0.870542
C	5.042078	-0.388950	-0.546321
O	5.173548	-1.695183	-0.840406
H	6.107737	-1.847200	-1.058096
O	5.973609	0.393515	-0.572512
Pd	-1.543157	-0.488082	0.575458
Cl	-2.820948	-2.397244	0.144764
O	-3.305171	0.542891	1.016959
H	-3.214162	1.535157	0.849000
H	-4.017485	0.198150	0.457204
Cl	-2.688632	3.415544	0.728504
O	-1.173227	2.317721	3.231252
H	-0.705728	2.913588	3.829625
H	-1.707610	2.885861	2.632345
O	-2.642320	-0.012049	3.816671
H	-2.125891	0.814831	3.774251
H	-3.127864	0.007427	2.979970

TS2

C	1.281990	-0.522931	0.216082
C	1.031545	0.819650	0.489496
C	2.071567	1.749149	0.444994
C	3.355295	1.317845	0.131415
C	3.604830	-0.033763	-0.139042
C	2.561686	-0.964395	-0.097597

H	1.871594	2.792840	0.657623
H	4.180504	2.017598	0.091583
H	2.749390	-2.010155	-0.307177
N	-0.326022	1.197752	0.832331
H	-0.399980	1.610492	1.791529
H	-0.697989	1.925221	0.212316
N	0.141917	-1.436931	0.271248
H	0.000058	-1.907705	-0.624679
H	0.276612	-2.168692	0.972485
C	4.999407	-0.440513	-0.464593
O	5.111568	-1.753606	-0.735796
H	6.046450	-1.927248	-0.933465
O	5.945360	0.324507	-0.488779
Pd	-1.569396	-0.460225	0.742670
Cl	-3.330356	-1.712183	-0.950813
O	-3.330814	0.539715	1.180700
H	-3.244181	1.524389	0.985028
H	-3.913856	0.155874	0.496314
Cl	-2.751832	3.413919	0.684514
O	-1.108622	2.435728	3.169377
H	-0.521165	3.091656	3.565758
H	-1.704946	2.947084	2.580611
O	-2.227422	-2.560599	1.666048
H	-2.732406	-2.463991	2.484857
H	-2.882485	-2.709554	0.952743

P

C	1.197842	-0.508199	0.168310
C	0.972782	0.830340	0.480144
C	2.012659	1.757055	0.410851
C	3.280492	1.324043	0.039545
C	3.511377	-0.024930	-0.257961
C	2.464396	-0.950919	-0.193555
H	1.828092	2.798028	0.648689
H	4.107695	2.019471	-0.025868
H	2.638057	-1.994230	-0.424921
N	-0.367403	1.201498	0.900086
H	-0.410372	1.459030	1.916689
H	-0.711435	2.039928	0.416599
N	0.053083	-1.409202	0.242416
H	-0.109225	-1.939669	-0.637891
H	0.189287	-2.104773	0.978295
C	4.890813	-0.430980	-0.643414
O	4.990541	-1.741842	-0.929817
H	5.916072	-1.913905	-1.168687
O	5.836413	0.333154	-0.697749
Pd	-1.658316	-0.353082	0.610697
Cl	-1.323248	-3.180205	-2.007835
O	-3.367515	0.784930	1.012211
H	-3.155757	1.773118	1.043031
H	-4.029557	0.685089	0.312114
Cl	-2.555998	3.632979	1.080044
O	-1.062763	2.145445	3.388709
H	-0.412838	2.666248	3.877418
H	-1.596189	2.801948	2.891125
O	-2.821145	-2.059257	0.265960
H	-3.753821	-1.868762	0.090563

H	-2.445876	-2.511400	-0.558829

b) [Pd(CPDA)Cl_2];

R

C	0.818010	-0.152967	0.280986
C	0.704995	1.158195	-0.184700
C	1.845975	1.864396	-0.558953
C	3.101203	1.257784	-0.469344
C	3.193726	-0.048503	0.009667
C	2.061492	-0.767891	0.395339
H	1.758258	2.877790	-0.932507
H	3.991368	1.793503	-0.773276
H	2.141598	-1.786016	0.754863
N	-0.632971	1.716875	-0.263831
H	-0.852990	2.210863	0.613355
H	-0.741292	2.381015	-1.045356
N	-0.419625	-0.840068	0.607223
H	-0.390668	-1.823142	0.342713
H	-0.644505	-0.787643	1.618283
Pd	-2.006826	0.145893	-0.294742
Cl	-3.707017	1.413853	-1.347618
Cl	-3.362541	-1.760035	-0.019123
O	-1.489990	3.381806	-2.467449
H	-1.630834	4.332393	-2.375193
H	-2.355734	2.962008	-2.295695
O	-1.692889	-0.314006	3.110862
H	-1.633296	0.658120	3.008272
H	-2.539866	-0.565889	2.714536
O	-1.214701	2.431065	2.569310
H	-0.467056	2.768764	3.081985
H	-1.936139	3.054175	2.733763
Cl	4.768602	-0.816010	0.121867

TS1

C	1.401241	-0.376348	-0.373837
C	1.152548	0.979139	-0.158951
C	2.195747	1.899480	-0.195793
C	3.493974	1.463080	-0.471447
C	3.721376	0.106734	-0.701021
C	2.688263	-0.830077	-0.648923
H	1.999547	2.953095	-0.031227
H	4.311819	2.170178	-0.522157
H	2.879247	-1.880377	-0.831358
N	-0.234274	1.364245	0.072988
H	-0.419989	1.489565	1.080286
H	-0.448571	2.242850	-0.396707
N	0.262339	-1.272419	-0.283211
H	0.329833	-2.054885	-0.931568
H	0.170266	-1.632266	0.688505
Pd	-1.467832	-0.174053	-0.586759
Cl	-3.608491	0.857642	0.825844

Cl	-2.619886	-2.052089	-1.354587
O	-2.806920	1.308240	-2.038420
H	-2.368557	2.122107	-2.321842
H	-3.386988	1.554294	-1.292036
O	-0.164924	-1.654131	2.487060
H	0.533540	-1.869068	3.116977
H	-0.504688	-0.767414	2.738507
O	-1.206952	0.915811	2.776893
H	-1.256960	1.426401	3.595248
H	-2.101389	0.939015	2.366085
Cl	5.342983	-0.441896	-1.083134

INT1

C	1.385224	-0.569097	0.077145
C	1.111991	0.729255	0.497794
C	2.129740	1.683257	0.518860
C	3.419154	1.332263	0.123568
C	3.673521	0.022218	-0.286882
C	2.669592	-0.943583	-0.313665
H	1.914472	2.695726	0.838348
H	4.214574	2.066665	0.130520
H	2.879729	-1.955364	-0.636525
N	-0.251749	1.042431	0.894879
H	-0.310422	1.307252	1.889419
H	-0.620760	1.854882	0.387874
N	0.277126	-1.523617	0.067251
H	0.190468	-1.982884	-0.840494
H	0.430971	-2.264606	0.753567
Pd	-1.479574	-0.587366	0.519235
Cl	-2.752172	-2.471690	-0.018266
O	-3.239503	0.424122	1.042189
H	-3.174693	1.414002	0.868390
H	-3.975733	0.067447	0.520407
Cl	-2.668424	3.333222	0.745075
O	-1.182127	2.373861	3.319647
H	-0.731564	2.976761	3.924674
H	-1.653423	2.929975	2.659377
O	-3.136030	0.452967	4.020498
H	-2.439948	1.132947	3.934489
H	-3.364357	0.250716	3.099929
Cl	5.295976	-0.423372	-0.778592

TS2

C	1.264715	-0.539653	0.214680
C	1.005813	0.797594	0.493700
C	2.047627	1.725765	0.483121
C	3.344677	1.306783	0.191120
C	3.581437	-0.040642	-0.088989
C	2.553240	-0.980416	-0.080689
H	1.847101	2.768475	0.701272
H	4.161324	2.017222	0.179078

H	2.749174	-2.022304	-0.301629
N	-0.363752	1.176233	0.806332
H	-0.424425	1.618802	1.745707
H	-0.716432	1.882266	0.155742
N	0.128540	-1.458391	0.237742
H	0.009367	-1.920114	-0.666419
H	0.254203	-2.197058	0.933901
Pd	-1.593083	-0.493862	0.693278
Cl	-3.327828	-1.733654	-1.068675
O	-3.369659	0.479094	1.131086
H	-3.312009	1.481127	1.057021
H	-3.950529	0.158061	0.415024
Cl	-2.983572	3.432461	0.996842
O	-0.836242	2.699114	3.149699
H	-0.182964	3.379458	3.357067
H	-1.543108	3.145721	2.633674
O	-2.259279	-2.634666	1.554569
H	-2.747150	-2.596981	2.388696
H	-2.917841	-2.783007	0.845338
Cl	5.208701	-0.565477	-0.472949

P

C	1.184566	-0.517705	0.152673
C	0.951486	0.809348	0.496229
C	1.994156	1.734775	0.473058
C	3.275862	1.319448	0.114966
C	3.493334	-0.020118	-0.213260
C	2.459815	-0.954620	-0.200754
H	1.809890	2.770084	0.735152
H	4.095499	2.026027	0.091705
H	2.640660	-1.989052	-0.463361
N	-0.400943	1.173410	0.899092
H	-0.429065	1.443968	1.903721
H	-0.735864	2.002939	0.399035
N	0.043746	-1.425090	0.187488
H	-0.094130	-1.939543	-0.702805
H	0.172041	-2.134631	0.911875
Pd	-1.676140	-0.387650	0.566711
Cl	-1.309165	-3.279242	-2.073817
O	-3.403570	0.720384	0.986136
H	-3.230273	1.709758	1.078926
H	-4.072728	0.633216	0.291021
Cl	-2.730194	3.599605	1.248105
O	-0.844564	2.394750	3.419956
H	-0.182114	2.972077	3.820052
H	-1.465623	2.981059	2.934903
O	-2.825439	-2.099071	0.180583
H	-3.762405	-1.926431	0.008585
H	-2.447496	-2.566253	-0.630005
Cl	5.105137	-0.541890	-0.662985

c) [Pd(hzpy)Cl_2]

<u>R</u>

C	-2.816467	1.150373	0.386899
C	-2.156821	-0.858254	-0.619676
C	-3.489081	-1.159640	-0.954945
C	-4.479319	-0.257893	-0.607471
C	-4.142734	0.923321	0.073079
H	-2.481262	2.033422	0.914386
H	-3.714045	-2.081422	-1.476099
H	-5.512151	-0.470951	-0.857247
H	-4.894123	1.648023	0.356673
N	-1.839491	0.276868	0.048803
N	-1.113982	-1.664208	-1.003115
H	-1.330999	-2.631385	-1.212384
N	0.130332	-1.467628	-0.329411
H	0.898623	-1.556006	-1.012587
H	0.242292	-2.177771	0.417098
Pd	0.149221	0.419819	0.497439
Cl	-0.007861	2.592761	1.414897
Cl	2.487487	0.281900	0.879262
O	2.903791	2.880077	2.831413
H	3.139604	2.055029	2.377349
H	1.990381	3.017728	2.524129
O	2.621191	-1.497581	-1.767129
H	2.919367	-0.840131	-1.111651
H	2.799433	-1.112020	-2.634286
O	0.254625	-3.449304	1.725400
H	0.393212	-4.375428	1.487571
H	0.870421	-3.273811	2.448972

<u>TS1</u>

C	-3.232646	2.227018	-0.032609
C	-3.371467	-0.113238	0.021373
C	-4.753100	-0.064156	-0.233099
C	-5.354120	1.172159	-0.388630
C	-4.586130	2.343297	-0.285770
H	-2.577064	3.081812	0.062242
H	-5.316707	-0.985693	-0.302491
H	-6.418590	1.229556	-0.583839
H	-5.027575	3.324126	-0.402499
N	-2.635180	1.021079	0.116222
N	-2.697632	-1.303450	0.125337
H	-3.223305	-2.135179	0.363223
N	-1.373536	-1.251797	0.644239
H	-0.758264	-1.820607	0.059558
H	-1.319710	-1.576786	1.630057
Pd	-0.679905	0.673007	0.566581
Cl	0.046052	2.892888	0.491702
Cl	1.609172	-0.565143	-0.548863
O	1.022066	0.255851	2.248451

H	1.659499	-0.073571	1.578936
H	1.336926	1.127390	2.530039
O	2.241082	-0.177522	-3.672524
H	2.079217	-0.280664	-2.714355
O	-0.599479	-1.735761	3.323380
H	-1.128839	-1.545816	4.107539
H	0.042161	-1.003721	3.236938
H	3.192993	-0.287411	-3.782883

INT1

C	-3.277854	1.260277	1.651660
C	-3.312569	-0.181290	-0.198392
C	-4.648512	-0.530172	0.065726
C	-5.283499	0.049745	1.148978
C	-4.591448	0.965092	1.958631
H	-2.685609	1.949810	2.236256
H	-5.150809	-1.241814	-0.576876
H	-6.313106	-0.207936	1.368536
H	-5.061445	1.435943	2.811472
N	-2.646733	0.697153	0.592361
N	-2.640447	-0.665238	-1.290735
H	-2.975539	-1.521644	-1.714427
N	-1.226049	-0.505372	-1.294248
H	-0.914665	-0.230650	-2.231041
H	-0.737013	-1.392001	-1.074168
Pd	-0.719306	0.969478	0.064459
Cl	-0.201286	2.679565	1.597113
Cl	1.500023	-0.261809	-3.193809
O	1.272322	1.092911	-0.552850
H	1.423565	0.757882	-1.487754
H	1.588757	2.006864	-0.492921
O	0.606438	-2.683139	-1.410575
H	1.090116	-2.075437	-2.011802
O	-2.829312	4.599510	1.419016
H	-2.631598	5.538736	1.516444
H	1.225947	-2.913357	-0.707074
H	-1.966412	4.148519	1.440519

TS2

C	-3.169018	1.044468	1.808384
C	-3.344144	-0.105527	-0.235644
C	-4.627883	-0.566888	0.106024
C	-5.163311	-0.190514	1.324223
C	-4.430080	0.635327	2.193444
H	-2.534509	1.660664	2.430526
H	-5.169177	-1.199365	-0.585476
H	-6.151035	-0.537647	1.603298
H	-4.827720	0.943634	3.150775
N	-2.638826	0.675600	0.618370
N	-2.767072	-0.369593	-1.446150
H	-3.082732	-1.175296	-1.971855
N	-1.388677	-0.043929	-1.594794
H	-1.276071	0.450690	-2.479219

H	-0.801608	-0.908910	-1.612970
Pd	-0.816601	1.166826	0.000271
Cl	0.296574	0.945057	2.536129
Cl	2.861709	-0.713764	-0.973644
O	1.080131	1.655941	-0.708035
H	1.691272	0.856269	-0.789908
H	1.067100	2.082827	-1.577118
O	0.282907	-2.263455	-1.735475
H	1.158196	-1.893993	-1.468776
O	-0.847123	3.280340	1.119018
H	-0.287363	3.912027	0.645986
H	0.110328	-3.007526	-1.144942
H	-0.306368	2.928117	1.858753

P

C	-3.043330	0.866627	1.876262
C	-3.341844	-0.043389	-0.267775
C	-4.633615	-0.483050	0.066635
C	-5.111960	-0.218491	1.337618
C	-4.307024	0.465682	2.263115
H	-2.364747	1.364634	2.558798
H	-5.223510	-1.016476	-0.667362
H	-6.104675	-0.552010	1.615828
H	-4.651032	0.674817	3.266644
N	-2.573329	0.622488	0.628949
N	-2.812266	-0.216651	-1.519912
H	-3.183134	-0.964081	-2.094685
N	-1.404609	-0.011313	-1.646086
H	-1.244511	0.493604	-2.517935
H	-0.887482	-0.923539	-1.689537
Pd	-0.744243	1.090735	-0.062680
Cl	-0.351408	1.455640	4.320081
Cl	2.818361	-0.876747	-1.313645
O	1.158775	1.534314	-0.817270
H	1.731458	0.718997	-0.993096
H	1.148773	2.056418	-1.633332
O	0.126502	-2.303894	-1.902897
H	1.033689	-1.972118	-1.699606
O	-0.121473	2.301927	1.513429
H	0.808779	2.515216	1.338094
H	-0.030514	-3.048966	-1.309245
H	-0.171594	1.962864	2.469080
